# Cationic amphiphilic drugs as potential anticancer therapy for bladder cancer

**DOI:** 10.1002/1878-0261.12793

**Published:** 2020-10-16

**Authors:** Geertje van der Horst, Arjanneke F. van de Merbel, Eline Ruigrok, Maaike H. van der Mark, Emily Ploeg, Laura Appelman, Siri Tvingsholm, Marja Jäätelä, Janneke van Uhm, Marianna Kruithof‐de Julio, George N. Thalmann, Rob C. M. Pelger, Chris H. Bangma, Joost L. Boormans, Gabri van der Pluijm, Ellen C. Zwarthoff

**Affiliations:** ^1^ Department of Urology Leiden University Medical Center The Netherlands; ^2^ Cell Death and Metabolism Center for Autophagy, Recycling and Disease Danish Cancer Society Research Center Copenhagen Denmark; ^3^ Department for BioMedical Research Urology Research Laboratory University of Bern Switzerland; ^4^ Department of Urology Inselspital Bern University Hospital University of Bern Switzerland; ^5^ Department of Urology Erasmus MC Cancer Institute Rotterdam The Netherlands; ^6^ Department of Pathology Erasmus MC Cancer Institute Rotterdam The Netherlands

**Keywords:** bladder cancer, cationic amphiphilic drugs, *ex vivo* culture, penfluridol, preclinical *in vivo* model

## Abstract

More effective therapy for patients with either muscle‐invasive or high‐risk non‐muscle‐invasive urothelial carcinoma of the bladder (UCB) is an unmet clinical need. For this, drug repositioning of clinically approved drugs represents an interesting approach. By repurposing existing drugs, alternative anticancer therapies can be introduced in the clinic relatively fast, because the safety and dosing of these clinically approved pharmacological agents are generally well known. Cationic amphiphilic drugs (CADs) dose‐dependently decreased the viability of a panel of human UCB lines *in vitro*. CADs induced lysosomal puncta formation, a hallmark of lysosomal leakage. Intravesical instillation of the CAD penfluridol in an orthotopic mouse xenograft model of human UCB resulted in significantly reduced intravesical tumor growth and metastatic progression. Furthermore, treatment of patient‐derived *ex vivo* cultured human UCB tissue caused significant partial or complete antitumor responses in 97% of the explanted tumor tissues. In conclusion, penfluridol represents a promising treatment option for bladder cancer patients and warrants further clinical evaluation.

## Graphical abstract

Repurposing FDA‐approved, antipsychotic cationic amphiphilic drugs like penfluridol dose‐dependently decreased viability of human bladder cancer cells, significantly reduced tumor progression and metastasis in an orthotopic human bladder cancer xenograft model, and induced significant antitumor responses in patient‐derived, *ex vivo* cultured bladder cancer tissue. Clinically approved penfluridol is a promising antineoplastic agent for bladder cancer patients for which effective therapy is currently lacking.

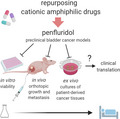

**Abbreviations**
CAD: cationic amphiphilic drug
MIBC: muscle‐invasive bladder carcinoma
NMIBC: non‐muscle‐invasive bladder carcinoma
TS: explanted tumor tissue slices
TURBT: transurethral resection of the bladder tumor
UCB: urothelial carcinoma of the bladder

## 1 Introduction

Urothelial carcinoma of the bladder (UCB) is the fifth most common cancer in the Western world [1]. Despite the prevalence and high economic costs of UCB, this cancer is still relatively understudied [2]. UCB presents either as non‐muscle‐invasive (NMIBC) or as muscle‐invasive carcinoma (MIBC), and many histopathological and molecular subgroups have been defined [3]. NMIBC patients undergo transurethral resection of the bladder tumor (TURBT), followed by adjuvant intravesical instillations with Bacillus Calmette–Guérin (BCG) in patients at high risk of recurrence and progression. However, patients do not always tolerate BCG and the risk of relapse in BCG‐treated patients varies between 30% and 40% [4].

Neoadjuvant cisplatin‐based chemotherapy and radical surgery are the recommended treatment options in nonmetastatic MIBC patients. Nevertheless, the 5‐year survival rate is only a mere 50% [5]. For patients with metastatic UCB, systemic cisplatin‐based chemotherapy is the standard of care [6].

Taken together, more effective therapies for high‐risk NMIBC and advanced or metastatic UCB patients are warranted.

By repurposing existing drugs, alternative anticancer therapies can be introduced in the clinic relatively fast, because the safety and dosing of these clinically approved pharmacological agents are generally well known. The risk of clinical failure due to (serious) adverse effects or reactions is, therefore, strongly reduced [7]. Consequently, the time needed for bringing the drug from bench to bedside is reduced [8].

Upon meta‐analysis, the overall risk ratio of cancer incidence was reduced among schizophrenia patients, although findings remain controversial [9, 10]. Strikingly, the incidence of UCB in patients with schizophrenia is significantly reduced when adjusted for differences in smoking prevalence [9]. Strikingly, a class of commonly used antidepressants, antihistamines, and antipsychotics—the so‐called cationic amphiphilic drugs (CADs)—were previously found to preferentially induce cell death in transformed cells [11]. The most potent CADs, including penfluridol, display preferential cytotoxicity toward transformed cells *in vitro* and potent antitumor activity even as single agents in murine tumor models (among others in breast, colon, pancreatic, and lung carcinoma) [11, 12, 13, 14, 15, 16, 17].

We hypothesized that CADs display antitumor activity in human UCB. We evaluated and compared the potential antitumor efficacy of multiple CADs in human UCB cell cultures *in vitro*, in a preclinical orthotopic human UCB growth and progression model, and in cultured, patient‐derived UCB tissue *ex vivo*.

## 2 Materials and methods

### 2.1 *In vitro*

Human UCB cell lines were cultured as described in Table S1 [18, 19].

#### 2.1.1 Cationic amphiphilic drugs

Penfluridol, astemizole, and terfenadine were obtained from Sigma‐Aldrich^®^ (Zwijndrecht, The Netherlands). Penfluridol (P3371) and terfenadine (T9652) were diluted in absolute EtOH (EMSURE^®^) to a stock solution of 50 mm. Likewise, astemizole (A2861), sertindole (S8072), chlorprothixene (C1671), chlorpromazine (C8138), clemastine (SML0445), and loratadine (L9664) were diluted in dimethyl sulfoxide (DMSO; Sigma‐Aldrich^®^) to a stock solution of 50 mm (Table S1). Serial dilutions were established by diluting these stock solutions in the cell‐specific medium of the various cell lines (Table S1).

#### 2.1.2 Viability

Cells were seeded as described previously [18]. Cells were seeded to reach subconfluency after 24 h and subsequently treated for 2 h with a dose range. After replacing the medium, cells were incubated at 37 °C for an additional 24, 48, or 72 h. Subsequently, 20 µL of MTS reagent was added, and after 2 h, the OD was measured at 490 nm using the VersaMax ELISA Microplate Reader (Molecular Devices, San Jose, CA, USA).

#### 2.1.3 Live cell count

Cell death was measured after staining for 15 min with propidium iodide (dead cells; 0.2 μg·mL^−1^) and Hoechst‐33342 (live cells; 2.5 μg·mL^−1^) at 37 °C employing Celígo^®^ Cytometer (Nexcelom Bioscience LLC., Lawrence, MA, USA) according to the manufacturer's manual.

#### 2.1.4 Apoptosis and necrosis

Cells were seeded in a 96‐well plate at a density of 10 000 single cells per well with three technical replicates. After 24 h, detection reagent (RealTime‐Glo™ Assay) was added. Subsequently, a dose range of penfluridol was added and luminescence and fluorescence were measured using SpectraMax iD3 (Molecular Devices, LLC., San Jose, CA, USA).

#### 2.1.5 Clonogenicity

Clonogenic assay was performed as described previously [18]. In brief, 100 single cells were seeded in a 6‐well plate. After 24 h, cells were treated with a dose range of penfluridol for 2 h. After replacing the medium, cells were incubated at 37 °C. After ~ 14 days, cells were fixed with 4% PFA and stained for 10 min with 5% crystal violet. The number of colonies and colony area were calculated with imagej (NIH, Bethesda, MD, USA) software.

#### 2.1.6 Lysosomal membrane permeabilization

60 000 cells were seeded on PET‐coated chamber slides and left for 24 h to adhere. Cells were treated with a dose range of penfluridol for 2 h. After 24 h, lysosomal membrane permeabilization was detected by staining paraformaldehyde‐fixed cells with LGALS1 and LAMP‐1 (Table S1). Confocal images were taken with TCS_SP8.

### 2.2 Orthotopic *in vivo* xenograft model

Female BALB/c nude mice (8 weeks old) were housed in ventilated cages under sterile conditions according to Dutch guidelines (evaluated by the Leiden Ethical Animal Welfare committee; DEC_14212; Table S1).

Human luciferase‐expressing UM‐UC‐3luc2 cells were inoculated into the bladder as described previously [19]. For both inoculation of healthy mice and the xenograft model, mice were intravesically treated weekly with either vehicle or penfluridol solution (100 µm equivalent to 130 µg·kg^−1^·week^−1^). The bladder was emptied by mild abdominal massage. Subsequently, 50 µL solution was inserted via an angiocatheter (24 G) and the urethra was closed for 1 h using a suture and thereafter followed by emptying of the bladder [19]. Bioluminescence imaging was performed using the IVIS Lumina [19]. Quantification of bioluminescent signals was performed by the living image ^®^ (Xenogen, PerkinElmer, Groningen, The Netherlands) software. Values were expressed as RLUs in photons/second. Numbers of metastases per animal were counted by eye from a dorsal and ventral view.

### 2.3 *Ex vivo* cultured UCB tissues

Tumor tissues from patients diagnosed with various stages of UCB were obtained during transurethral resection of the bladder upon informed consent (evaluated by the Erasmus Medical Ethical Committee MEC‐2014‐553) and conform the standards set by the Declaration of Helsinki. Explanted tumor tissue slices (TS) were sectioned and cultured as previously described [20, 21].

#### 2.3.1 Histology and immunofluorescence staining

Hematoxylin and eosin staining (H&E) was performed according to a standard protocol to assess general histology. For immunofluorescence staining, deparaffinization was performed via incubation in Histo‐Clear (National Diagnostics, Atlanta, GA, USA, HS‐200) and rehydrated by incubation in a series of decreasing concentrations of ethanol. Antigen retrieval was performed by cooking the slides in unmasking solution (Vector Labs, Burlingame, CA, USA, H‐3300) in the pressure cooker. After blocking with 1% BSA (Sigma‐Aldrich, A7906) for 30 min, the slides were incubated overnight with primary antibody at 4 °C. Next, the slides were stained for 1.5 h at RT with secondary antibody and mounted with ProLong Gold Antifade (Molecular Probes, Thermo Fisher scientific, Breda, The Netherlands, P36930) (Table S1).

#### 2.3.2 Scoring and inclusion criteria

Upon fixation, paraffin embedding, and sectioning, TS were stained for H&E, apoptosis (c‐CASP‐3), proliferation (PCNA), and cytokeratins (KRT) [21]. Stained sections were scanned with a slide scanner and evaluated with CaseViewer. Immunohistochemical evaluations were based on visual estimations from complete histological tissue sections. Confocal images were taken from representative regions.

Criteria for the inclusion of UCB tissue are as follows: (a) diagnosis of UCB in TS is confirmed by the pathology report; (b) majority of directly fixed and/or vehicle‐treated TS contain tumor cells; and (c) size of obtained material is sufficient to perform at least vehicle and 1 CAD dosage. First, H&E‐stained TS were scored for overall quality (score 0: good quality; 1: poor tissue integrity and quality in > 50% of TS area; 4: tissue completely degraded or fragmented). Then, sections were scored for the presence of apoptosis (c‐CASP‐3^+^ tumor cells) and fragmented cytokeratin (loss of cell integrity) (score 0: sporadic; 1: multiple clusters). Finally, TS were scored for the presence of PCNA (score 1: < 50% of KRT+ cells displayed nuclear PCNA). Total score: cumulative individual scores (Fig. S4).

### 2.4 Statistics

Analysis was performed using Graphpad Prism 7.0 (Graphpad software, San Diego, CA, USA) and Excel; for *in vitro* assays, one‐way ANOVA and Bonferroni *post‐hoc* test; for *in vivo assays,* Mann–Whitney *U*‐tests; and for *ex vivo assays*, chi‐squared test for categorical data with more than two categories. *P < 0.05;**P < 0.01;***P < 0.001.

## 3 Results

### 3.1 Cationic amphiphilic drugs induce lysosomal‐dependent cell death *in vitro*

**Fig. 1:**
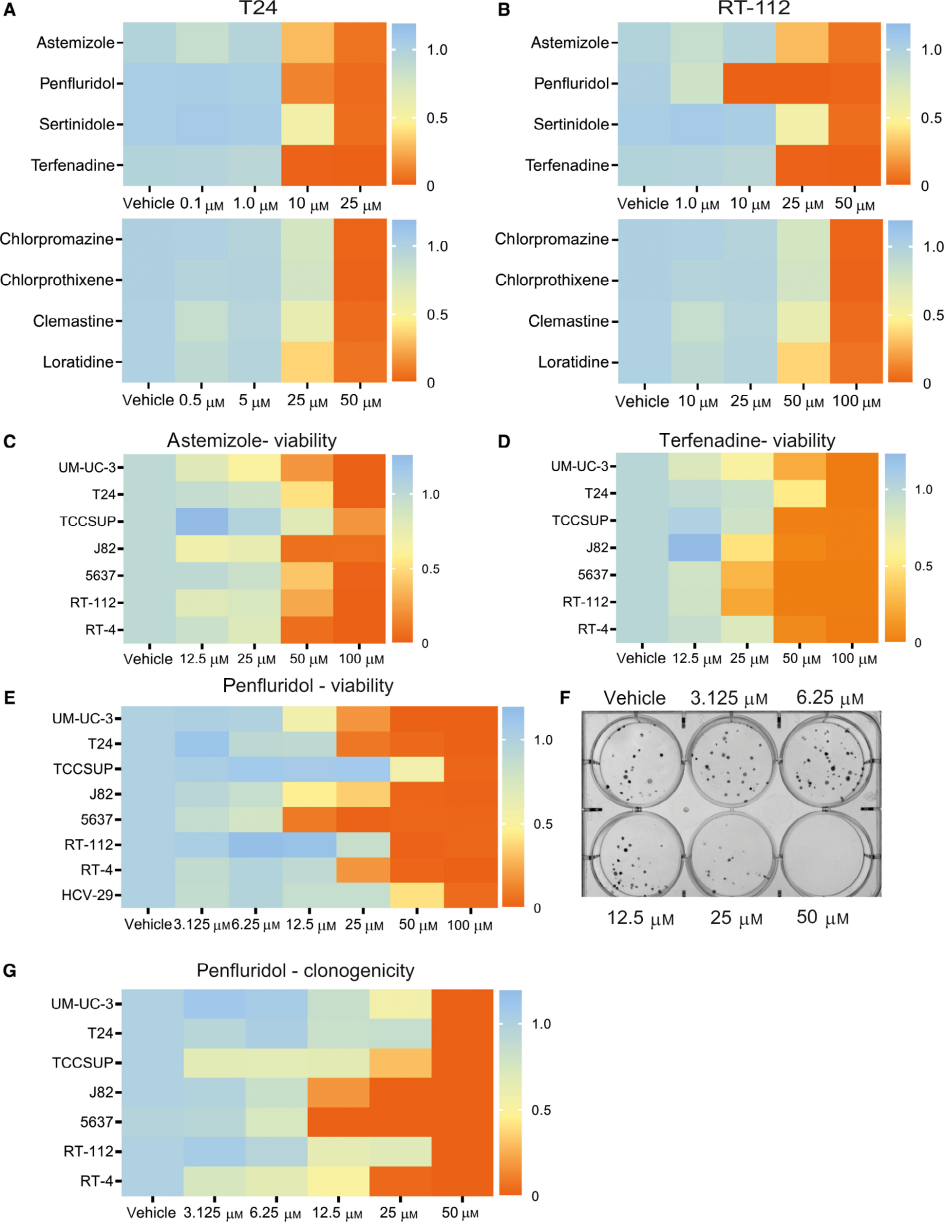
CADs reduce viability and clonogenicity in a panel of human bladder cancer cells. Assessment of the viability of T24 (A) and RT‐112 (B) cells after treatment with eight different CADs for 40 h (*n* = 3; six replicates each). Panels C–E represent dose–response experiments with astemizole, terfenadine, and penfluridol, respectively, on the viability of multiple subconfluent bladder cancer cells. Viability was measured 48 h after a 2‐h treatment. Mean normalized to vehicle‐treated cells (*n* = 3; six replicates each). Clonogenic assay: Multiple bladder cancer cells were treated for 2 h with a dose range of penfluridol. (F) Representative image of a clonogenic assay UM‐UC‐3 cells. (G) The number of colonies was measured using imagej after 10–14 days of culture (*n* = 3; three replicates each).

To identify CADs that display antitumor effects, 8 CADs were selected—at least in part—based on a library screen on lung cancer and on the NCI drug sensitivity database [22]. These CADs were initially tested for their effect on viability of T24 and RT‐112 human UCB cells *in vitro*. The CADs penfluridol, astemizole, and terfenadine induced the strongest antitumor effects upon short‐term exposure (Fig. 1A,B; Fig. S1A–D). Subsequently, short‐term exposure to a dose range of these CADs also led to a dose‐dependent inhibition in viability of a panel of UCB cells (Fig. 1C–E; Fig. S1E–G). Viability was decreased when a confluent layer of UCB cells was exposed to a dose range of penfluridol (Fig. S1J). Furthermore, penfluridol significantly decreased the clonogenicity of multiple UCB cell lines (Fig. 1F,G; Fig. S2A–D). Since we observed strongest antitumor potential with penfluridol, this compound was used in other disease models *in vivo* and *ex vivo*.

**Fig. 2:**
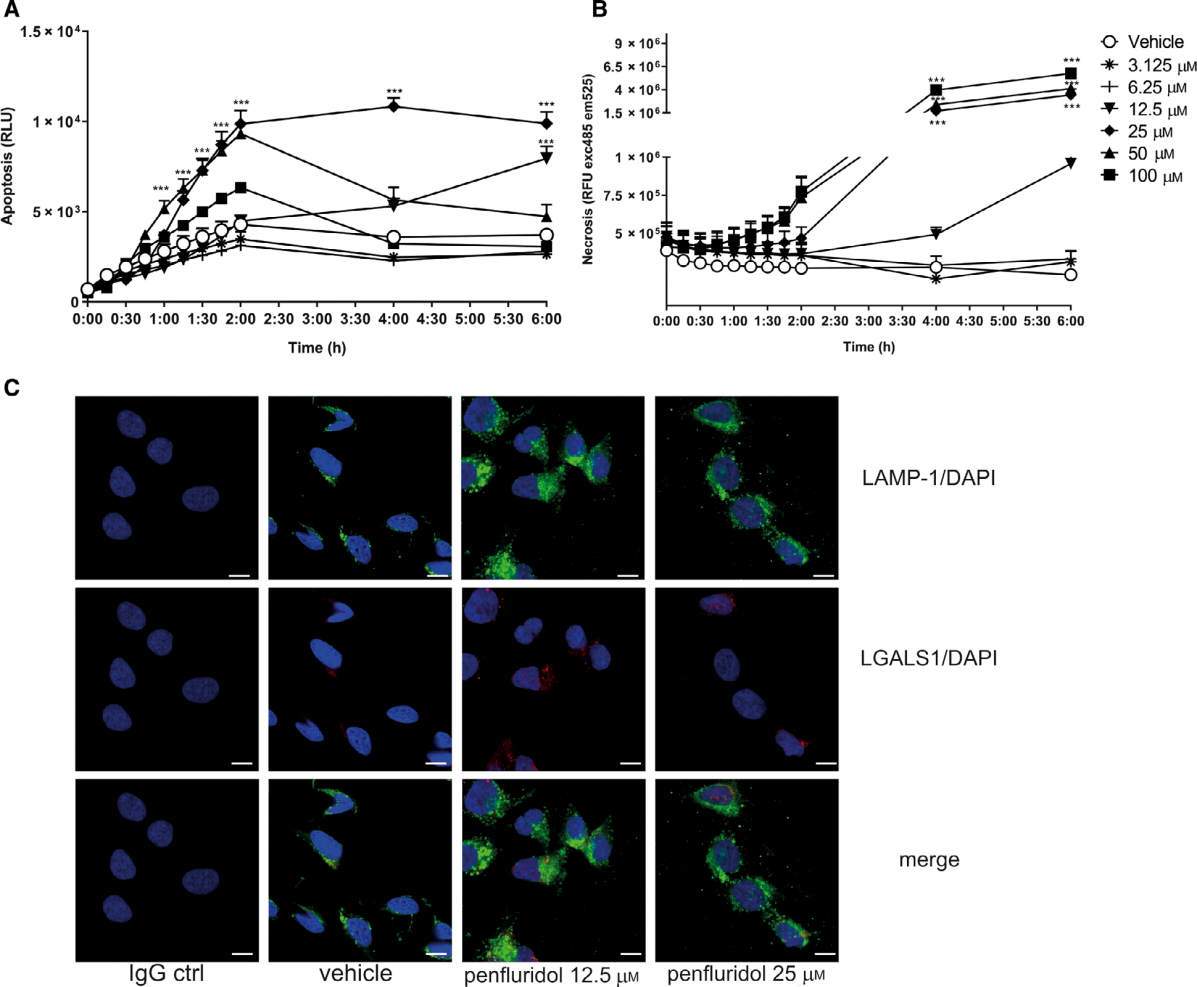
Penfluridol induces cell death in human bladder cancer cells. (A) Real‐time apoptosis (luminescence measurement of phosphatidylserine on outer leaflet of cell membranes with annexin V) and (B) cell death (fluorescence measurement of a DNA‐binding dye) after administration of a dose range of penfluridol. Data presented are mean ± SE (*n* = 3; six replicates each). One‐way ANOVA. *P < 0.05; **P < 0.01; ***P < 0.001. (C) Penfluridol targets lysosomal structures in UM‐UC‐3 cells (*n* = 3, 3 replicates each). Representative images of LAMP‐1 (green)‐ and LGALS1 (red)‐/DAPI (blue)‐stained UM‐UC‐3 cells treated for 2 h with a dose range of penfluridol.

Penfluridol treatment resulted in redistribution of phosphatidylserine from the internal to external membrane surface, an early indicator of apoptosis. This was followed by lysis of the cell as measured with a DNA‐binding dye (Fig. 2A,B). For the higher dosage (100 µm), only a small increase in apoptosis was observed, and already loss of membrane integrity was observed within 2 h. Furthermore, lysosomal LGALS1 puncta formation, a hallmark of lysosomal leakage, was found in penfluridol‐treated cells (Fig. 2C) [11, 12, 23, 24].

### 3.2 Penfluridol inhibits UCB growth and metastasis in a preclinical orthotopic xenograft model

**Fig. 3:**
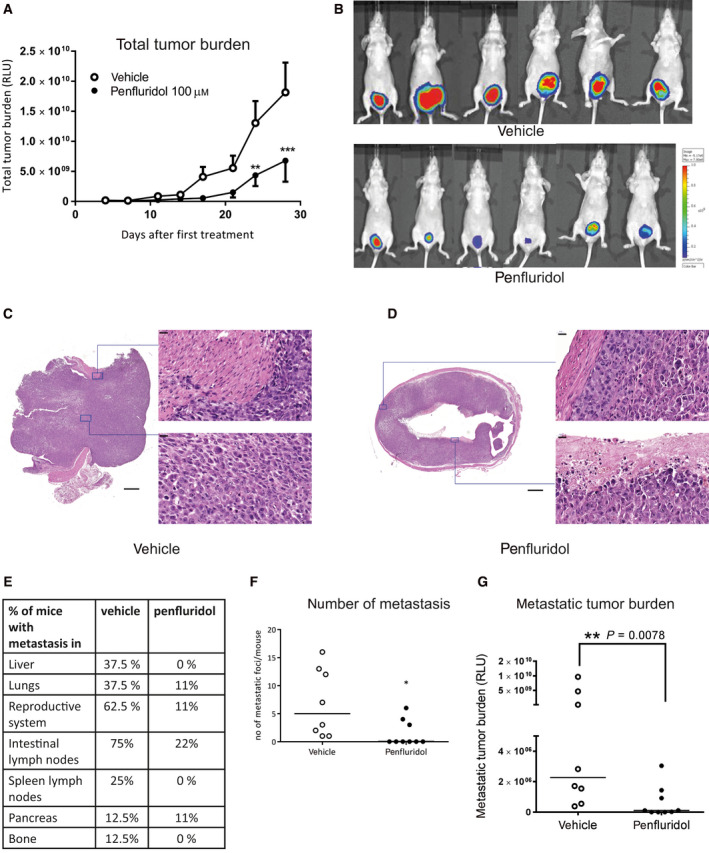
The antitumor effects of penfluridol in an orthotopic murine xenograft model with stable *firefly luciferase‐2* UM‐UC‐3 human bladder cancer cells. Female BALB/c nude mice were inoculated with *firefly luciferase‐2‐*labeled UM‐UC‐3 bladder cancer cells and intravesically treated with vehicle (*n* = 8) or penfluridol (*n* = 9; 100 µm once weekly; equivalent to 130 µg·kg^−1^·week^−1^). (A) Quantification of the whole‐body total tumor burden in real time (relative light units, RLU). Data are presented as mean ± SE. Mann–Whitney *U*‐test. *P < 0.05; **P < 0.01; ***P < 0.001. (B) Representative bioluminescent images of vehicle‐ and penfluridol‐treated mice at day 29. Representative images of vehicle (C)‐ and penfluridol (D)‐treated bladders stained with hematoxylin and eosin. Scale bar: 500 and 20 µm, respectively. (E) The percentage of mice with detectable metastases in the listed organs. (F) Number of metastatic foci per mouse per experimental group. (G) Metastatic tumor burden per experimental group (RLU). Mann–Whitney *U*‐test. *P < 0.05; **P < 0.01; ***P < 0.001.

In order to evaluate the antitumor effects of penfluridol in a preclinical xenograft mouse model, *firefly luciferase‐*2‐expressing human UM‐UC‐3luc2 cells were inoculated in the bladder of female immunodeficient mice (BALB/c nude) [19]. Four days after tumor cell inoculation, tumor burden was assessed by whole‐body bioluminescence imaging (BLI) and properly inoculated mice were equally distributed among two groups with similar median tumor burden (Fig. S3A,B) [19]. At this stage, bladder tumor cells have not yet invaded the muscle layer of the murine bladder. Subsequently, the mice were treated by weekly intravesical instillations of penfluridol or vehicle solution. Penfluridol significantly reduced total tumor burden by 63% (Fig. 3A,B). No significant changes were observed in body weight (Fig. S3C). At day 29, mice were sacrificed and size and weight of the bladders of penfluridol‐treated mice were diminished compared with vehicle‐treated animals (Fig. S3D,E). As expected, histological examination revealed large orthotopically growing tumors in vehicle‐treated mice with invasion of the tumors into the surrounding connective tissue and muscle layers (Fig. 3C). In contrast, in penfluridol‐treated mice no tumor invasion into the bladder muscle layer was observed and large necrotic regions in tumor tissues at the luminal side of the bladder were detected (Fig. 3D). All vehicle‐treated mice developed metastatic disease with an average of 6.8 metastases/mouse, while only 33% of the penfluridol‐treated animals developed distant metastases with an average of 4.3/mouse (Fig. 3E). Metastases were observed in the lungs, reproductive system, liver, intestinal or mesenteric lymph nodes, pancreas, bone, and spleen lymph nodes (Fig. S3F). Both the number of metastases/mouse and metastatic tumor burden were significantly decreased in penfluridol‐treated mice (Fig. 3F,G).

### 3.3 Antineoplastic response of penfluridol in *ex vivo* cultured, patient‐derived cancer tissue slices

Next, we examined the antineoplastic effect of penfluridol in our ‘near‐patient’ model, that is, *ex vivo* cultured patient‐derived UCB tissue slices [21]. Tumor tissues from 57 patients diagnosed with different stages of bladder carcinoma were obtained during transurethral resection of the bladder (TURB) procedures. Tumor tissues from 39/57 patients (68%) were included according to a predefined set of tissue quality criteria (see Materials and methods: Histology and immunofluorescence staining; Table 1).

**Table 1:** Patient characteristics. Tumor tissues from 57 patients diagnosed with various stages of UCB were obtained during transurethral resection of the bladder (TURBT upon informed consent, MEC‐2014‐553). Tumor tissues from 39 patients were included according to the tissue quality inclusion criteria. Disease stage, grade, and presence of CIS and detrusor were determined by a pathologist. Patient #40 is a patient diagnosed with prostate carcinoma Gleason grade 3 + 4 with no history of UCB. NA, not applicable; BCG, Bacillus Calmette–Guérin.

Patient no	Disease stage	Disease grade	Tumor	Recurrence frequency		Cis present	Detrusor present	Previous treatment	Previous treatment modality	Gender	Age
1	Ta	G1	Primary	NA		Unknown	Yes	No	NA	Male	75
2	Ta	G1	Primary	NA		Unknown	Yes	No	NA	Male	71
3	Ta	G1	Recurrence	> 1/year	Unknown		Yes	Unknown	Unknown	Male	72
4	Ta	G1	Primary	NA		Unknown	Yes	No	NA	Female	69
5	Ta	G1	Primary	NA		Unknown	No	No	NA	Male	62
6	Ta	G1	Recurrence	> 1/year		Unknown	No	Yes	Nephroureterectomies	Male	63
7	Ta	G2	Primary	NA		Unknown	Yes	No	NA	Male	76
8	Ta	G2	Primary	NA		No	Yes	No	NA	Male	67
9	Ta	G2	Recurrence	> 1/year		No	Yes	Yes	TURBT only	Male	86
10	Ta	G2	Recurrence	> 1/year		No	Yes	Yes	Chemotherapy	Male	56
11	Ta	G2	Recurrence	> 1/year		No	Yes	Yes	BCG	Male	87
12	Ta	G2	Recurrence	> 1/year		No	Yes	Yes	Nephroureterectomies	Male	62
13	Ta	G2	Primary	NA		No	Unknown	No	NA	Male	39
14	Ta	G2	Recurrence	> 1/year		No	Yes	Yes	BCG	Male	59
15	Ta	G2	Primary	NA		No	Yes	No	NA	Female	69
16	Ta	G2	Recurrence	> 1/year		No	Yes	Yes	Chemotherapy	Female	53
17	Ta	G2	Recurrence	> 1/year		No	Yes	Yes	Chemohyperthermia	Male	57
18	Ta	G2	Primary	NA		No	Yes	No	NA	Male	79
19	Ta	G2	Primary	NA		No	Yes	No	NA	Male	73
20	Ta	G2	Recurrence	< 1/year		No	Yes	Yes	Chemotherapy	Male	75
21	Ta	G2	Recurrence	> 1/year		No	Yes	Yes	BCG	Female	59
22	Ta	G2	Recurrence	Unknown		No	Yes	Yes	Chemoradiation	Male	84
23	Ta	G3	Primary	NA		No	Yes	No	NA	Male	75
24	Ta	G3	Recurrence	> 1/year		No	No	Yes	BCG	Male	76
25	Ta	G3	Primary	NA		Unknown	No	No	NA	Male	56
26	Ta	Unknown	Primary	< 1/year		No	Unknown	No	NA	Female	53
27	T1	G2	Recurrence	< 1/year		Unknown	No	No	NA	Male	86
28	T1	G2	Recurrence	> 1/year		No	Yes	Yes	BCG	Male	74
29	T1	G2	Primary	NA		Yes	Yes	No	NA	Male	67
30	T1	G2	Primary	NA		No	Yes	No	NA	Male	55
31	T1	G3	Primary	NA		No	Yes	Yes	TURBT only	Male	66
32	T1	G3	Recurrence	> 1/year		No	Yes	Yes	BCG	Male	52
33	T1	G3	Primary	NA		No	No	No	NA	Female	67
34	T1	G3	Primary	NA		Unknown	Yes	No	NA	Male	81
35	T2	G3	Primary	NA		No	Yes	No	NA	Male	74
36	T2	G3	Primary	NA		Yes	Yes	No	NA	Male	58
37	T2	G3	Recurrence	> 1/year		Yes	Yes	No	NA	Male	72
38	T2	G3	Primary	NA		Unknown	Yes	No	NA	Male	66
39	T2	High grade	Primary	NA		Unknown	Yes	No	NA	Male	64
40	T3	Gl3 + 4	Primary	NA		NA	NA	Yes	LHRH agonist	Male	65

Tumor tissues were treated with vehicle solution or a dose range of penfluridol (*n* = 18) for 3 days. When tumor biopsy material was limited (*n* = 21), explanted tumor tissues were cultured in the presence of vehicle solution or 100 µm penfluridol for 3 days.

**Fig. 4:**
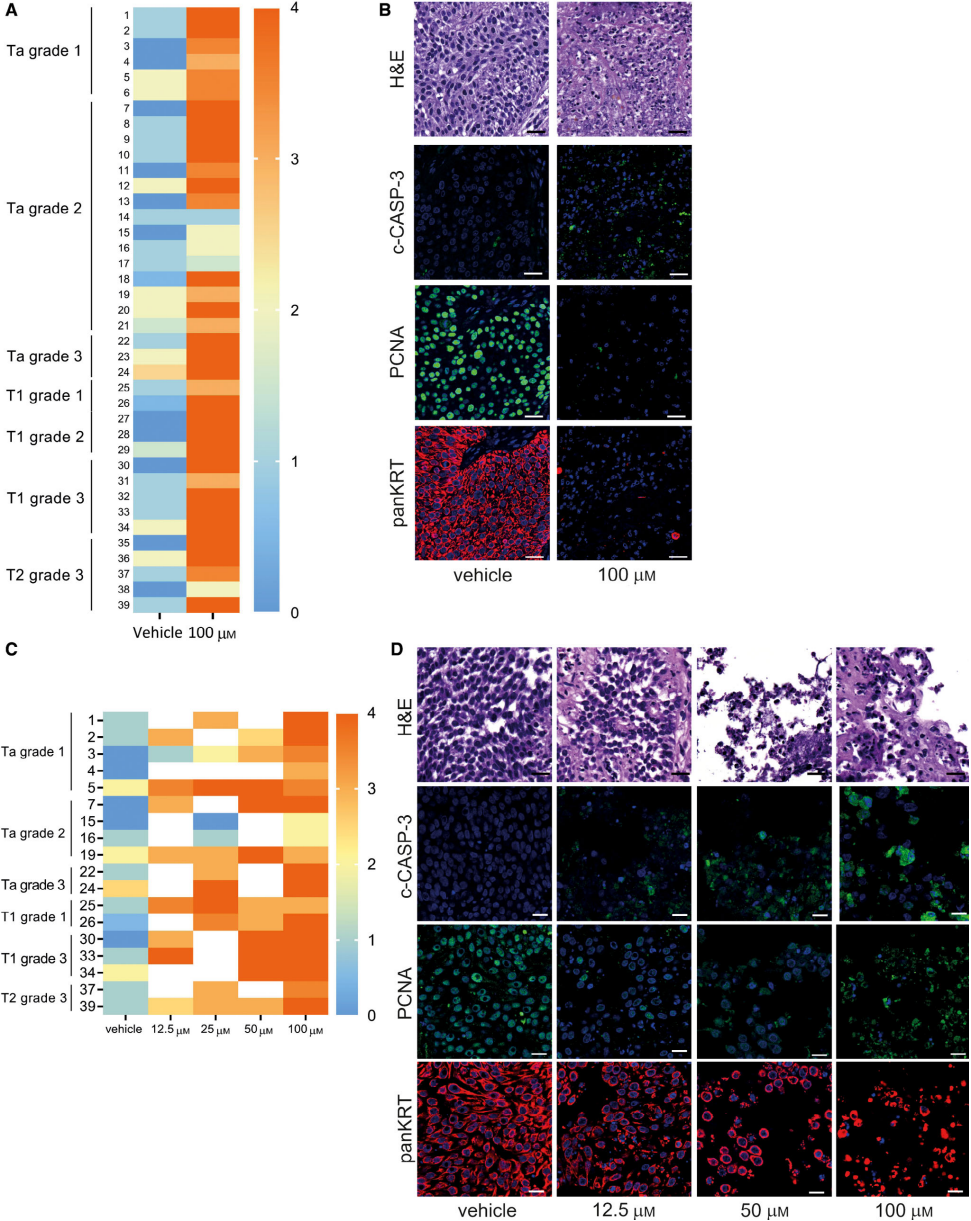
*Ex vivo* treatment of cultured human bladder cancer slices with penfluridol. Heat maps showing median cumulative scores of the bladder cancer slices per condition per patient. Multiple explanted tissue slices were cultured for each condition. Cumulative score is based on histological evaluation of entire tissue slice (TS; see Fig S4). TS were scored in four categories: (1) overall quality based on H&E staining, (2) the presence of apoptotic cells [cleaved caspase 3^+^(c‐CASP‐3^+^)/keratin^+^ (KRT^+^) cells], (3) cancer cell debris (fragmented keratin), and (4) nuclear proliferation based on proliferation cell nuclear antigen (PCNA). TS received a score of either 0 or 1 for each category. For category 1, TS received a score of 1 when > 50% of tissue was fragmented/degraded. For categories 2 and 3, TS received a score of 1 when multiple clusters were observed. For category 4, TS received a score of 1 when < 50% of KRT+ cells in the TS displayed nuclear PCNA. Cumulative score was calculated as the sum of the 4 categories; median cumulative scores are shown (see also Fig. S4). Explanted tumor slices were *ex vivo* cultured with vehicle solution or penfluridol (100 µm for A–B and a dose range for C–D) for 3 days. Chi‐squared test for categorical data with more than two categories. *P* value = 4.36424E^−11^. (B) Representative images of bladder cancer slices obtained from a patient diagnosed with NMIBC stage T1 grade 2 (#28) after 3 days in the presence of vehicle solution or penfluridol (100 µm). Complete loss of tumor cells was observed in five patient‐derived tumor explants (#9, 28, 29, 31, and 35). Significant reduction was found in 30 patient‐derived tumor explants (#1–8, 10–15, 17, 20, 22–27, 30, 32–34, and 36–39). A partial response was observed in 3 patient‐derived tumor explants (#18, 19, and 21). No response was observed in one patient‐derived tumor explant (#16). (D) Representative images of bladder cancer tissue slices obtained from a patient diagnosed with NMIBC stage T1 grade 2 (#9) after 3 days in the presence of vehicle solution or a dose range of penfluridol. c‐CASP‐3 and PCNA: green; panKRT: red; and DAPI: blue. Scale bar: 25 µm.

Culturing of the tumor tissue slices under control conditions only marginally affected tissue integrity and viability or not at all, as indicated by marginal differences in immunohistochemical localization of fragmented cytokeratin (indicative of cancer cell debris), apoptotic cells (c‐CASP‐3), and proliferating cells (PCNA) compared to directly fixed tissue (scoring method in Fig. S4 and results in Fig. S5). In almost all patients (35/39), strong changes were observed after treatment with penfluridol, ranging from complete loss of tumor to significant reduction of the tumor (Fig. 4A). An overall reduction in the number of UCB cells, proliferating cells (PCNA), increased numbers of apoptotic cells (c‐CASP‐3), and significantly increased amounts of fragmented cytokeratins were observed (*P* value = 4.36424E^−11^, Χ^2^ test). In tissue slices obtained from only three patients, partial antineoplastic responses were registered (e.g., an induction of apoptosis). In tissue slices obtained from one patient (1/39), no response to the treatment was found. The effect of a dose range of penfluridol revealed that, starting from 12.5 µm, major antineoplastic changes were observed (Fig. 4C,D).

### 3.4 Penfluridol has no effect on normal urothelium

**Fig. 5:**
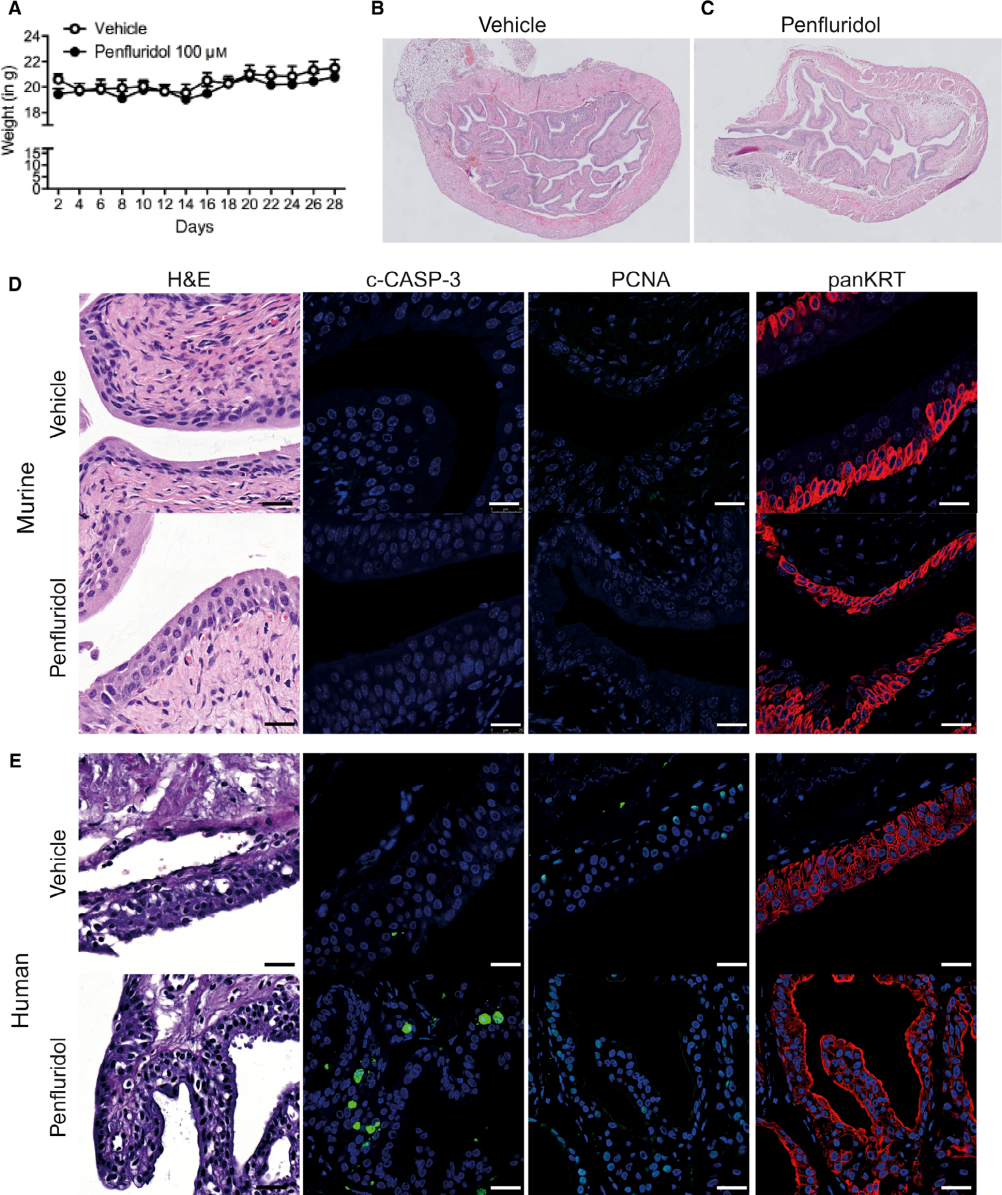
Evaluation of the effect of penfluridol on normal murine or human urothelium integrity. (A) Average body weight (mean ± SE) of non‐tumor‐bearing female BALB/c nude mice treated intravesically with vehicle (*n* = 5) or penfluridol solution (*n* = 5; 100 µm once weekly for 4 weeks; equivalent to 130 µg·kg^−1^·week^−1^). One‐way ANOVA. *P < 0.05; **P < 0.01; ***P < 0.001. Representative overview images of H&E‐stained murine bladders of vehicle‐treated (B) and 100 µm penfluridol‐treated (C) mice. (D) Representative images of vehicle‐ and penfluridol‐treated, non‐tumor‐bearing murine bladders. c‐CASP‐3 and PCNA: green; panKRT: red; and DAPI: blue. Scale bar: H&E: 20 µm; IF staining: 25 µm. (E) Representative images of nontransformed ‘normal’ urothelium in tissue slices obtained from a patient with prostate cancer with no history of bladder carcinoma, treated either with vehicle solution or with 100 µm penfluridol for 3 days (#40). c‐CASP‐3 and PCNA: green; panKRT: red; and DAPI: blue. Scale bar: 25 µm.

Intravesical treatment represents a potential alternative route of administration of CADs for the treatment of localized bladder cancer. In order to evaluate safety of penfluridol instillations on normal urothelium, a dose range of penfluridol was administered to non‐tumor‐bearing mice by weekly intravesical treatment (Fig. 5A). Histological analyses revealed no detectable changes of normal murine urothelium after multiple doses of intravesical penfluridol (Fig. 5B–D). Moreover, while penfluridol treatment of *ex vivo* cultured patient‐derived UCB tissues caused significant antitumor effects, adjacent ‘normal’ human urothelium was not or only marginally affected (Fig. S6A,B). No changes were observed in epithelial tissue integrity, the proliferation index (PCNA), viable epithelial cell numbers, or fragmented KRT, despite a mildly increased apoptotic response (Fig. S6A). Because matched normal urothelium samples are scarce, we assessed the effect of penfluridol on ‘normal’ urothelium of a prostate cancer patient with no history of bladder carcinoma. Again, no detectable tissue integrity changes were observed in the penfluridol‐treated human urothelium versus vehicle‐treated urothelium. Limited c‐CASP‐3^+^ cells were only observed in the penfluridol‐treated normal urothelium layers, but no detectable changes of urothelium tissue integrity were found (Fig. 5E).

## 4 Discussion

In this study, we provide compelling evidence that CADs display significant antitumor effects in multiple preclinical bladder cancer models, that is, cultured human UCB cells *in vitro*, an orthotopic preclinical xenograft model of UCB growth and metastasis *in vivo*, and patient‐derived *ex vivo* cultured UCB tissues. Screening of eight different CADs in a panel of cultured human UCB cell lines identified penfluridol as a potent candidate anticancer agent. We observed that the antitumor properties of penfluridol in human UCB may rely on rapid destabilization of lysosomal structures in human UCB cells.

Our data in UCB are in line with the observed antineoplastic effects of CADs in other solid tumors [11, 12, 13, 14, 15, 16, 17]. Other antineoplastic mechanisms of this class of compounds may also play a role, including antimigratory and invasion effects, cell cycle effects, changes in cholesterol homeostasis, autophagy, immune modulation, or nonhomologous end joining (reviewed in {Shaw, 2019 #2068} [25, 26, 27, 28].

Cationic amphiphilic drugs have been shown to induce lysosome‐dependent cell death mediated by lysosomal cathepsin proteases leaking from the destabilized lysosomes to the cytosol in other tumors [29, 30, 31]. Selectivity of the CADs for transformed cells is due to the lower constitutional acid sphingomyelin levels in their lysosomes compared to healthy cells. CADs reduce ASM levels to the point of lysosomal membrane permeabilization which leads to lysosome‐dependent cell death specifically in transformed cells [29, 30, 32]. Selectivity of CADs for transformed bladder epithelial cells is further substantiated by the observed lack of adverse effects of CAD treatment on normal urothelium, which was not (or only marginally) affected after intravesical administration of a same dose of penfluridol in a non‐tumor‐bearing *in vivo* model and administration of penfluridol to *ex vivo* cultured normal human bladder tissue.

In our preclinical disease model of orthotopically growing human UCB, weekly intravesical instillation with penfluridol significantly reduced tumor progression, invasion, and formation of distant metastases. Our preclinical data demonstrate that effective delivery of penfluridol can be achieved by instillation in the bladder, thus mimicking the clinical use of standard‐of‐care pharmacological agents mitomycin C or BCG. This way, potential adverse systemic side effects of this class of antipsychotic drugs may be largely avoided.

For UCB patients that present with a disease confined to the mucosa (stage Ta, CIS) or submucosa (stage T1), treatment consists of transurethral resection of the bladder (TURB) followed by an immediate postoperative intravesical instillation with chemotherapy. In patients who are at intermediate or high risk of recurrence, maintenance treatment with intravesical chemo‐ or immunotherapy is advocated in the guidelines. In general, chemotherapy with mitomycin C or epirubicin for 1 year (4 weekly instillations followed by 6–8 monthly instillations) is indicated in intermediate‐risk patients, whereas high‐risk patients are treated with 1 to 3 years of Bacillus Calmette–Guérin (BCG) [33]

Patients in whom NMIBC recurs after TURB and BCG treatment, and who have BCG‐unresponsive disease, are unlikely to respond to further BCG therapy and may need a cystectomy. The alternative treatment option is lifelong cystoscopic surveillance, and frequent TURB re‐operations are necessary. Obvious downsides of radical cystectomy are that it is a major surgical procedure with substantial cost and a negative impact on the quality of life of patients. If feasible, patients often prefer a bladder sparing treatment and new intravesical therapies are being developed in clinical trials [34].

Despite these new developments, only valrubicin—a doxorubicin analog—was FDA‐approved for BCG‐refractory CIS [35]. Based on the growing body of evidence and the previously established safety profile of CADs as FDA‐approved drugs, penfluridol appears to be a promising anticancer compound, especially when systemic exposure is reduced by intravesical administration to patients with (recurrent) high‐risk bladder cancer. Taken together, our current findings emphasize the need for a clinical trial that will address safety and antitumor efficacy aspects of intravesical penfluridol in organ‐confined bladder cancer.

## 5 Conclusions

Cationic amphiphilic drugs like penfluridol significantly reduced UCB cell viability. Weekly bladder instillations of penfluridol resulted in significant decreased tumor growth, progression, and metastasis *in vivo*. Treatment of cultured patient‐derived UCB tissue slices with penfluridol resulted in significant antitumor responses ranging from complete loss of tumor to significant reduction. Normal urothelium, when treated with penfluridol, was not at all or only marginally affected upon instillation of CADs in a preclinical UCB *in vivo* model and cultured normal human bladder tissue slices *ex vivo*.

Taken together, penfluridol is a promising anticancer agent in human UCB and clinical studies are required to determine whether this FDA‐approved antipsychotic agent can provide a benefit to patients with UCB.

## Conflict of interest

The authors declare no conflict of interest.

## Author contributions

ECZ, GP, GH, and MJ conceived the study. MKJ, GH, MJ, MM, and AM contributed to methodology. GH, AM, ST, MJ, GP, and ECZ performed formal analysis. GH, AM, ER, MM, EP, LA, ST, and MJ participated in investigation. GH wrote the original draft of the manuscript. GP, JB, ECZ, RP, CB, and GH reviewed and edited the manuscript. GH, GP, ECZ, and CB acquired funding. JB, GT, JU, RP, and MKJ collected resources. GP and ECZ supervised the study.

## Supporting information


**Fig. S1.** CADs reduced viability in a panel of human bladder cancer cells.
**Fig. S2.** CADs reduced viability and clonogenicity in a panel of human bladder cancer cells.
**Fig. S3.** Anti‐tumor effects of penfluridol in an orthotopic murine xenograft model with stable firefly luciferase‐2 UM‐UC‐3 human bladder cancer cells.
**Fig. S4.** Scoring of ex‐vivo cultured human bladder cancer slices.
**Fig. S5.** Ex‐vivo treatment of cultured human bladder cancer slices with penfluridol: directly fixed vs cultured tissue.
**Fig. S6.** Evaluation of the effect of penfluridol on normal human urothelium.
**Fig. S7.** Evaluation of the effect of penfluridol on normal murine urothelium.

**Table S1.** Key resources.

**Appendix S1.** Supplementary materials and methods.
