# Supplementary material for: Cationic amphiphilic drugs as potential anticancer therapy for bladder cancer
Source: Mol Oncol. 2020 Oct 16;14(12):3121–34. doi: 10.1002/1878-0261.12793 (PMC7718956; doi:10.1002/1878-0261.12793)
Supplement: Supplementary file 1 — Fig. S1. CADs reduced viability in a panel of human bladder cancer cells. Fig. S2. CADs reduced viability and clonogenicity in a panel of human bladder cancer cells. Fig. S3. Anti‐tumor effects of penfluridol in an orthotopic murine xenograft model with stable firefly luciferase‐2 UM‐UC‐3 human bladder cancer cells. Fig. S4. Scoring of ex‐vivo cultured human bladder cancer slices. Fig. S5. Ex‐vivo treatment of cultured human bladder cancer slices with penfluridol: directly fixed vs cultured tissue. Fig. S6. Evaluation of the effect of penfluridol on normal human urothelium. Fig. S7. Evaluation of the effect of penfluridol on normal murine urothelium. [file MOL2-14-3121-s001.pdf]

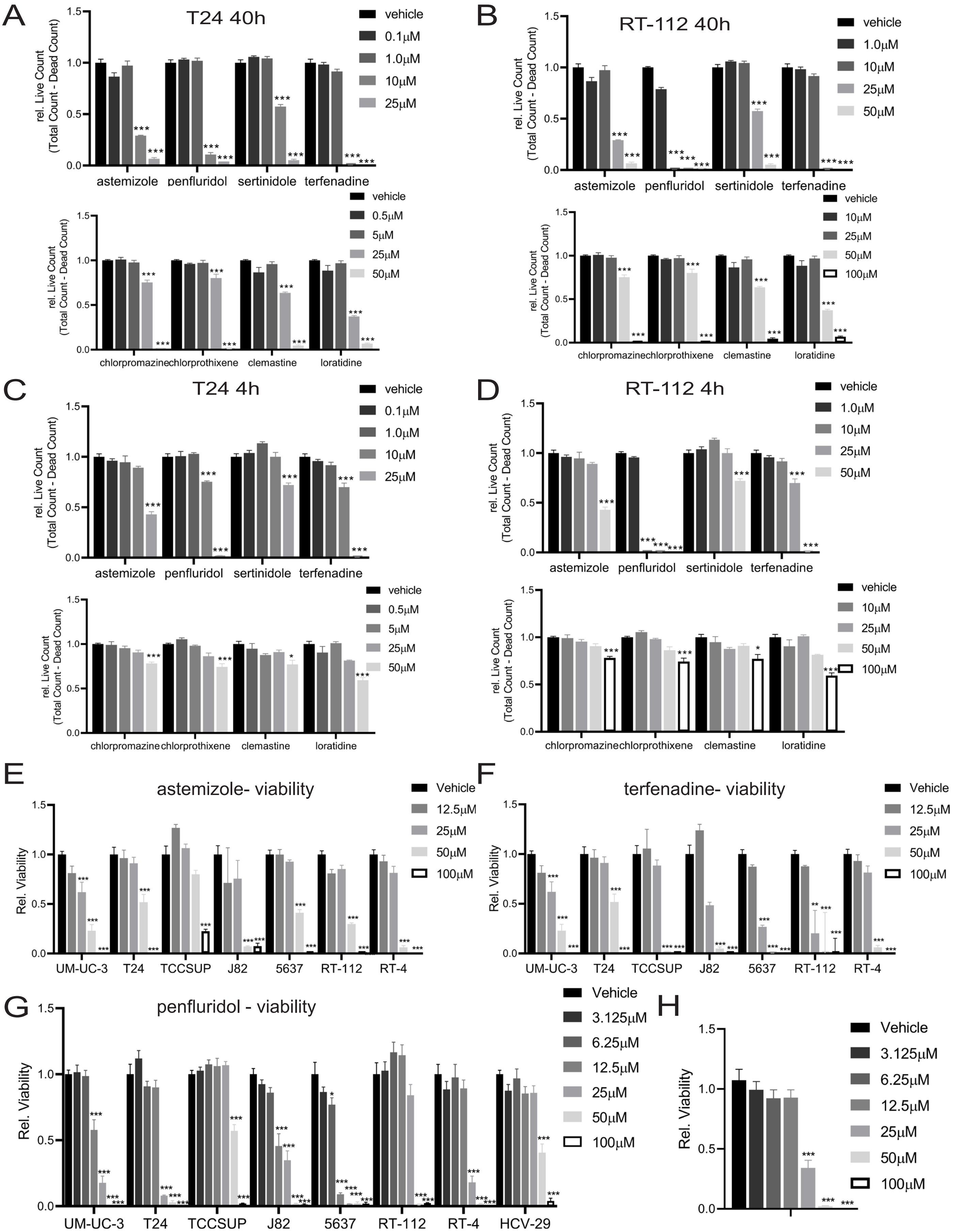

Figure S1 CADs reduced viability in a panel of human bladder cancer cells.
Assessment of the viability of T24 (A) and RT-112 (B) cells after treatment with a dose range of 8 different CADs for 40h. Assessment of the viability of T24 (C) and RT-112 (D) cells after 4hrs treatment. CADs were replaced by regular growth medium after 4h and 40h later live cell count was quantified in a Celigo(R) cytometer as the total cell count (Hoechst-positive cells) - dead cell count (Propidium Iodide- positive cells). Mean +/- SE normalized to vehicle treated cells. One-way ANOVA *p<0.05; **p<0.01; ***p<0.001 Multiple subconfluent UCB cells were treated for 2h with a dose range of either astemizole (E), terfenadine (F) or penfluridol (G). Viability was measured using MTT assay after 48h. Mean +/- SE normalized to respective vehicle treated UCB cells. H) Confluent UM-UC-3 cells were treated for 2h with a dose range of penfluridol. Viability was measured using MTT assay after 48h. Mean +/- SE normalized to vehicle treated cells. (n=3; 6 replicates each). One-way ANOVA *p<0.05; **p<0.01; ***p<0.001.

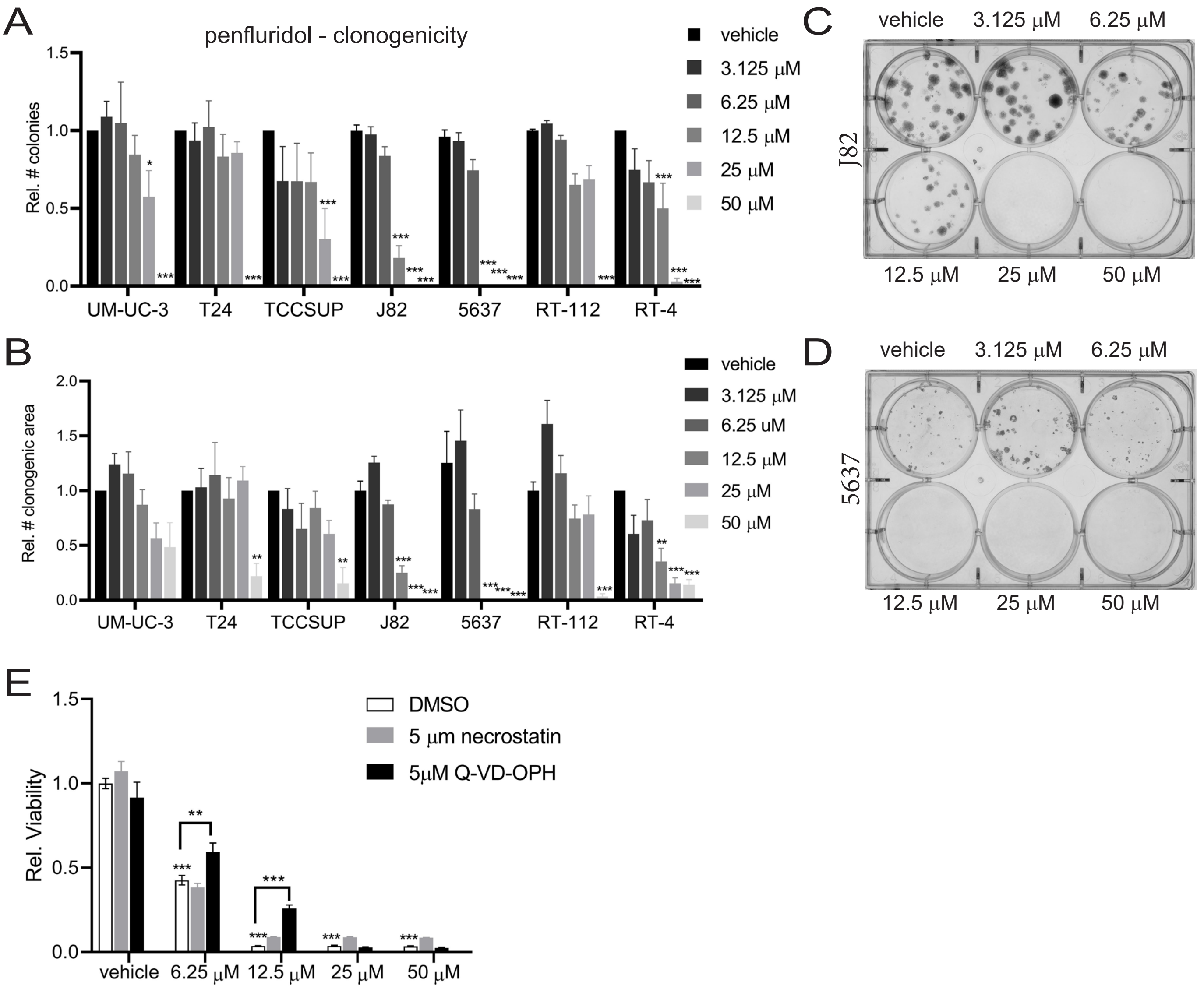

Figure S2 CADs reduced viability and clonogenicity in a panel of human bladder cancer cells.
Clonogenic assay: multiple bladder cancer cells were treated for 2h with a dose range of penfluridol and subsequently after 10-14 days, A) number of colonies and B) clonogenic area were measured using Image J. Representative images of clonogenic assay of respectively J82 (C) and 5637 (D) cells. (n=3; 3 replicates each). One-Way ANOVA. *$p<0.05$; **$p<0.01$; ***$p<0.001$. E) Mitochondrial activity was measured after 24h in response to treatment with a dose range of penfluridol in combination with either necroptosis inhibitor NS1 or pan-caspase inhibitor Q-VD-OPh. Mean+/- SE normalized to vehicle treated UM-UC-3 cells (n=3; 6 replicates each). One-way ANOVA. *$p<0.05$; **$p<0.01$; ***$p<0.001$.

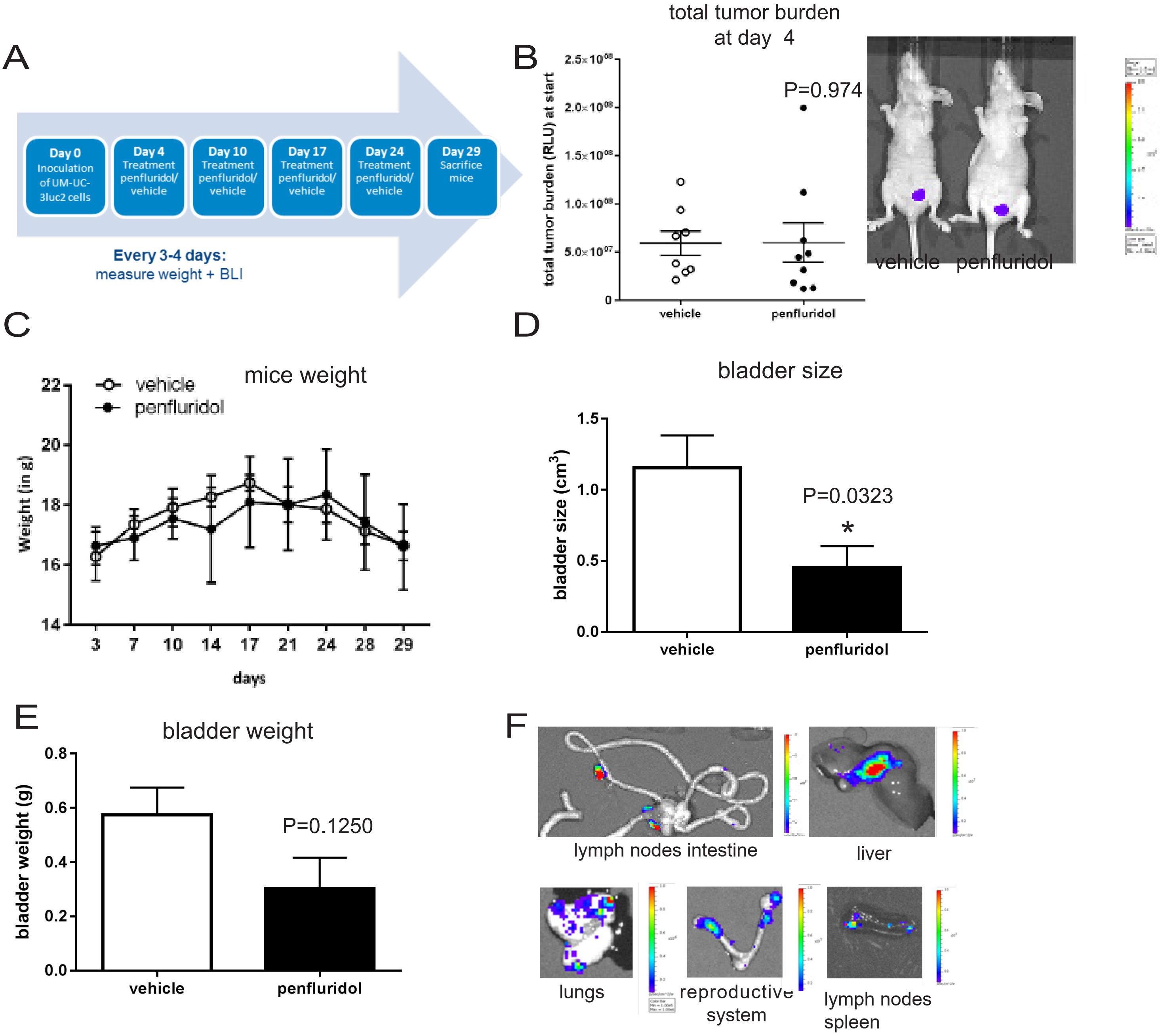

Figure S3 Anti-tumor effects of penfluridol in an orthotopic murine xenograft model with stable *firefly luciferase-2* UM-UC-3 human bladder cancer cells.

Female balb C nu/nu mice were inoculated with UM-UC-3 luciferase 2 bladder cancer cells and intravesically treated with vehicle (n=8) or penfluridol (n=9; 100µM once weekly; equivalent to 130 µg/kg/w). A) Schematic overview of the experiment. B) mice were divided into 2 groups with equal tumor burden based on total tumor burden; representative BLI images of mice are shown. C) weight of the mice over time. D) bladder size in cm^3 at day 29. E) bladder weight in g at day 29. F) representative BLI images of metastasis in intestinal lymph nodes, liver, spleen, reproductive system, and lungs at day 29. Mann-Whitney-U test. *p<0.05; **p<0.01; ***p<0.001.

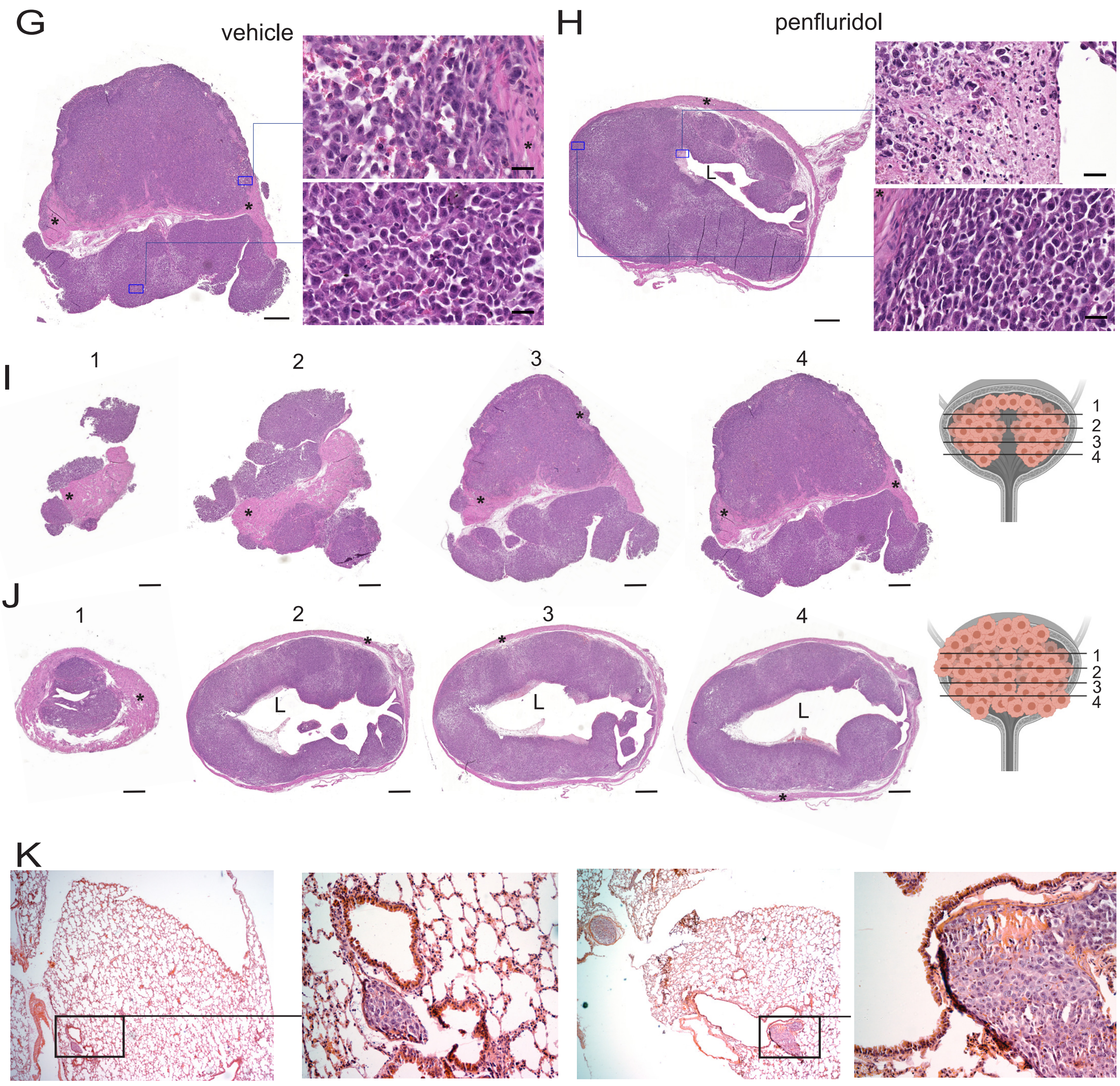

Figure S3 Anti-tumor effects of penfluridol in an orthotopic murine xenograft model with stable *firefly luciferase-2* UM-UC-3 human bladder cancer cells.

Representative images of vehicle (G) and penfluridol (H) treated bladders stained with Hematoxylin & Eosin with respective magnifications. Scale bar 500 and 20 µm respectively. I) Representative images of vehicle treated bladders stained with Hematoxylin & Eosin at different planes of sectioning displaying muscle-invasion. Scale bar 500 µm.Schematic overview of the bladders is shown next to the different planes (created with Biorender.com).
J) Representative images of penfluridol treated bladders stained with Hematoxylin & Eosin at different planes of sectioning. Scale bar 500 µm. K) representative Hematoxylin & Eosin images of a metastasis in a lung.
L: lumen; * muscle layer.

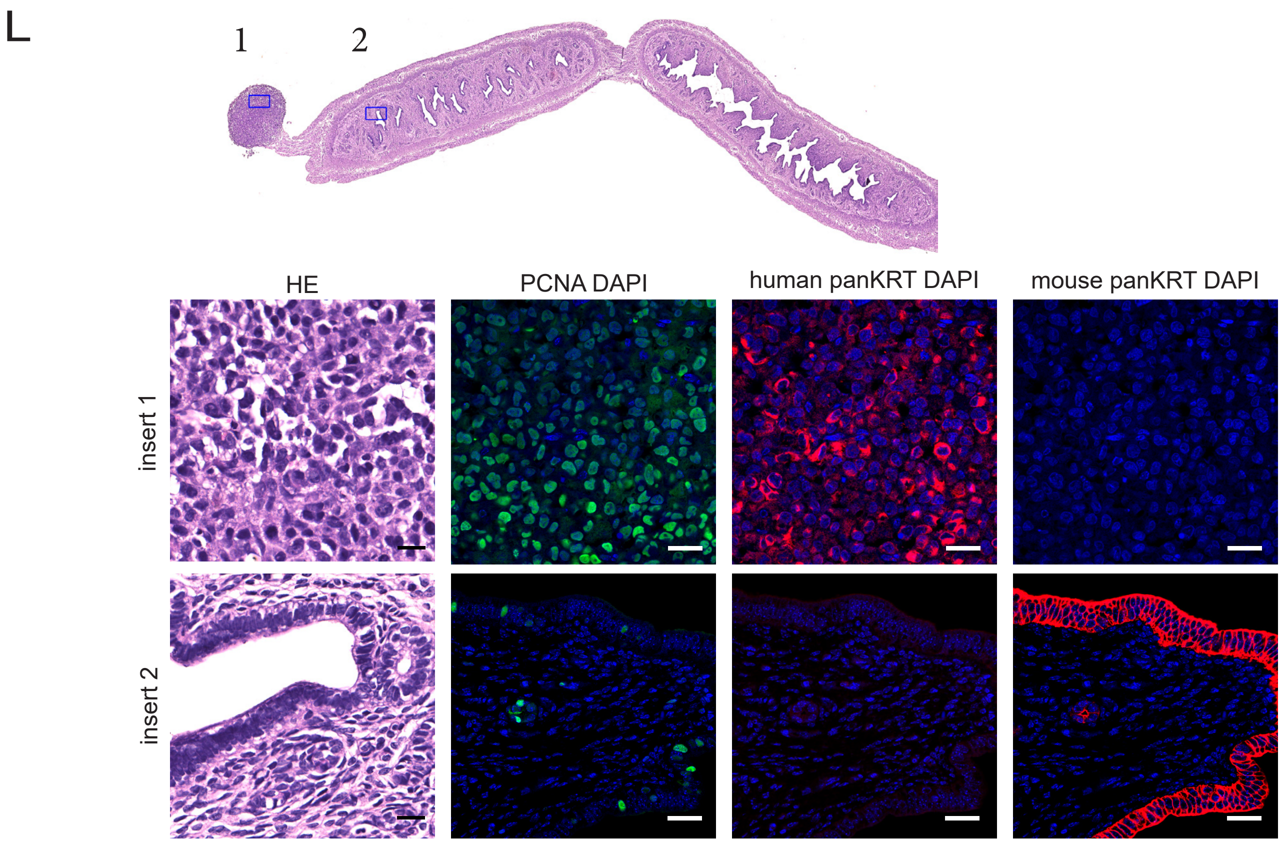

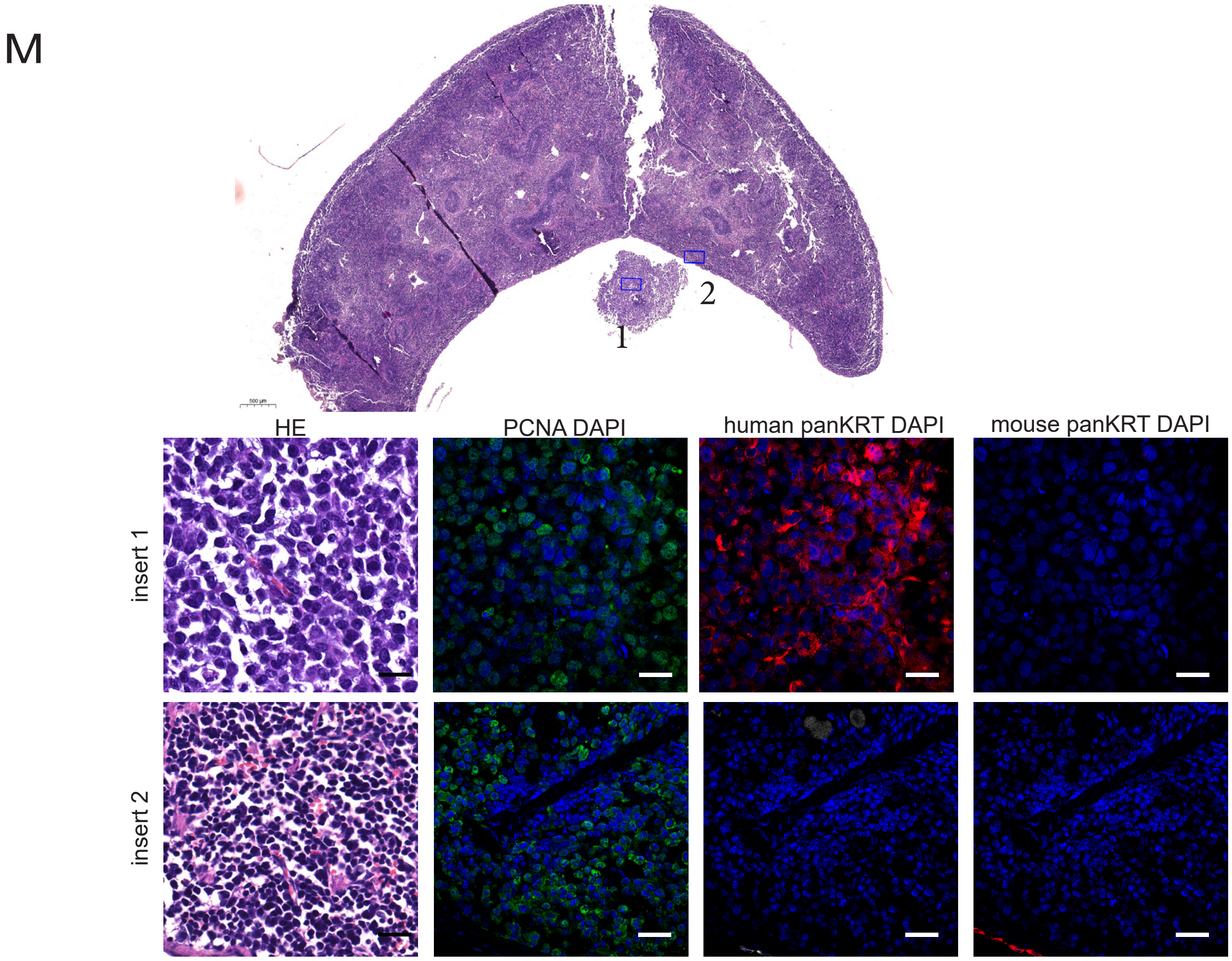

Figure S3 Anti-tumor effects of penfluridol in an orthotopic murine xenograft model with stable *firefly luciferase-2* UM-UC-3 human bladder cancer cells.
L) representative image of a metastasis in a lymph node (adjacent to spleen). M) representative image of a metastasis in the reproductive system. Scale bar for overview 500μm. PCNA: green, panKRT (human or mouse): red and DAPI blue. Scalebar 25μm.

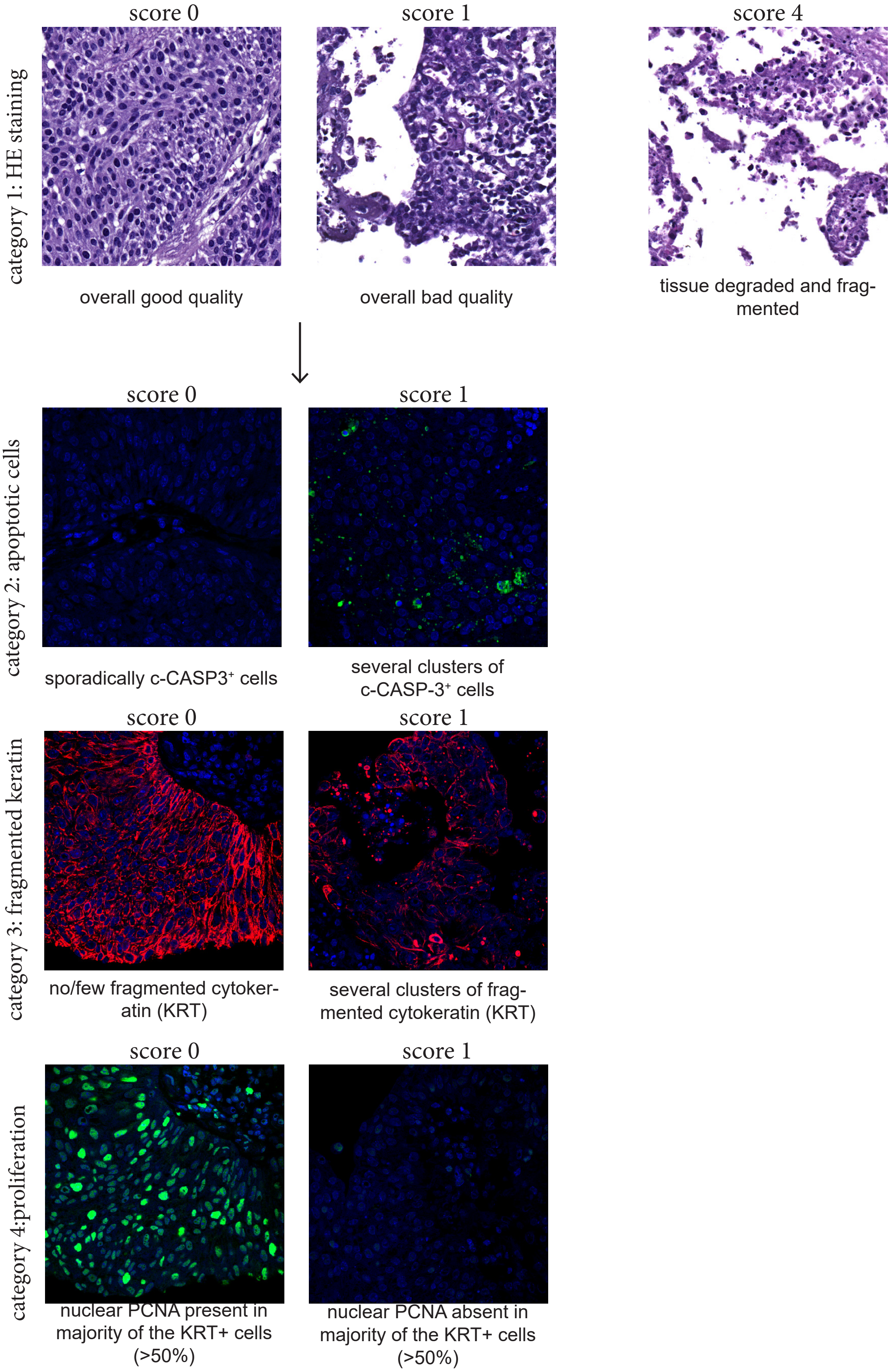

Figure S4 ***Scoring of ex-vivo* cultured human bladder cancer slices**
Multiple explanted tissue slices were cultured for each condition. Cumulative score is based on histological evaluation of entire tissue slice. Representative images are shown. First, overall quality of the TS was scored based on H&E staining (category 1). TS received a score of either 0, 1 or 4. TS received a score of 1 when overall quality was poor in >50% of the H&E stained TS. The TS scored the maximum score of 4 when tissue was completely degraded or fragmented. When TS did not already directly receive a score of 4 for overall quality based on the H&E stained tissue, sections were subsequently scored for 3 additional categories: 2) presence of cleaved-caspase 3+(c-CASP-3+)/Keratin+ (KRT+) cells, 3) fragmented KRT, and 4) nuclear proliferation based on proliferation cell nuclear antigen (PCNA). TS received a score of either 0 or 1 for each category. For category 2 and 3, TS received a score of 1 when multiple clusters were observed. For category 4, TS received a score of 1 when <50% of KRT+ cells in the TS displayed nuclear PCNA. Cumulative score was calculated as the sum of the 4 categories, median cumulative scores are shown. c-CASP-3 and PCNA: green, panKRT red and DAPI blue. Scalebar 25μm.

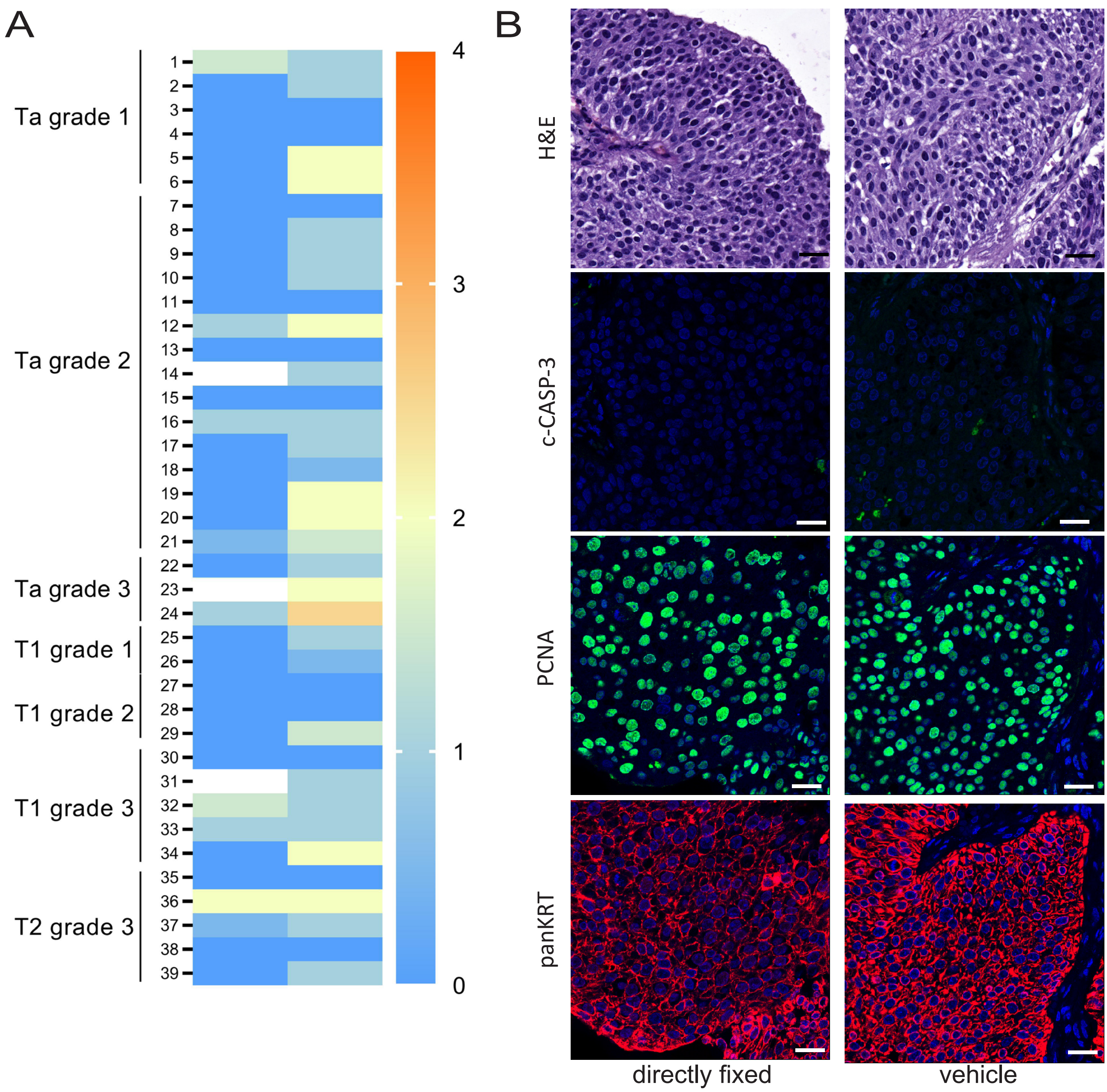

Figure S5 Ex-*vivo* treatment of cultured human bladder cancer slices with penfluridol: directly fixed vs cultured tissue

A) heat map showing the median scores of the bladder cancer slices per patient (sum of the scores based on PCNA, c-CASP-3, fragmented KRT and H&E staining). Bladder cancer slices from directly fixed and *ex vivo* cultured in vehicle solution for 3 days. B) representative images of bladder cancer tissue obtained from a patient diagnosed with NMIBC stage T1 grade 2 (#28) directly fixed or cultured for 3 days in the presence of vehicle solution. c-CASP-3 and PCNA: green, panKRT red and DAPI blue. Scalebar 25µm.

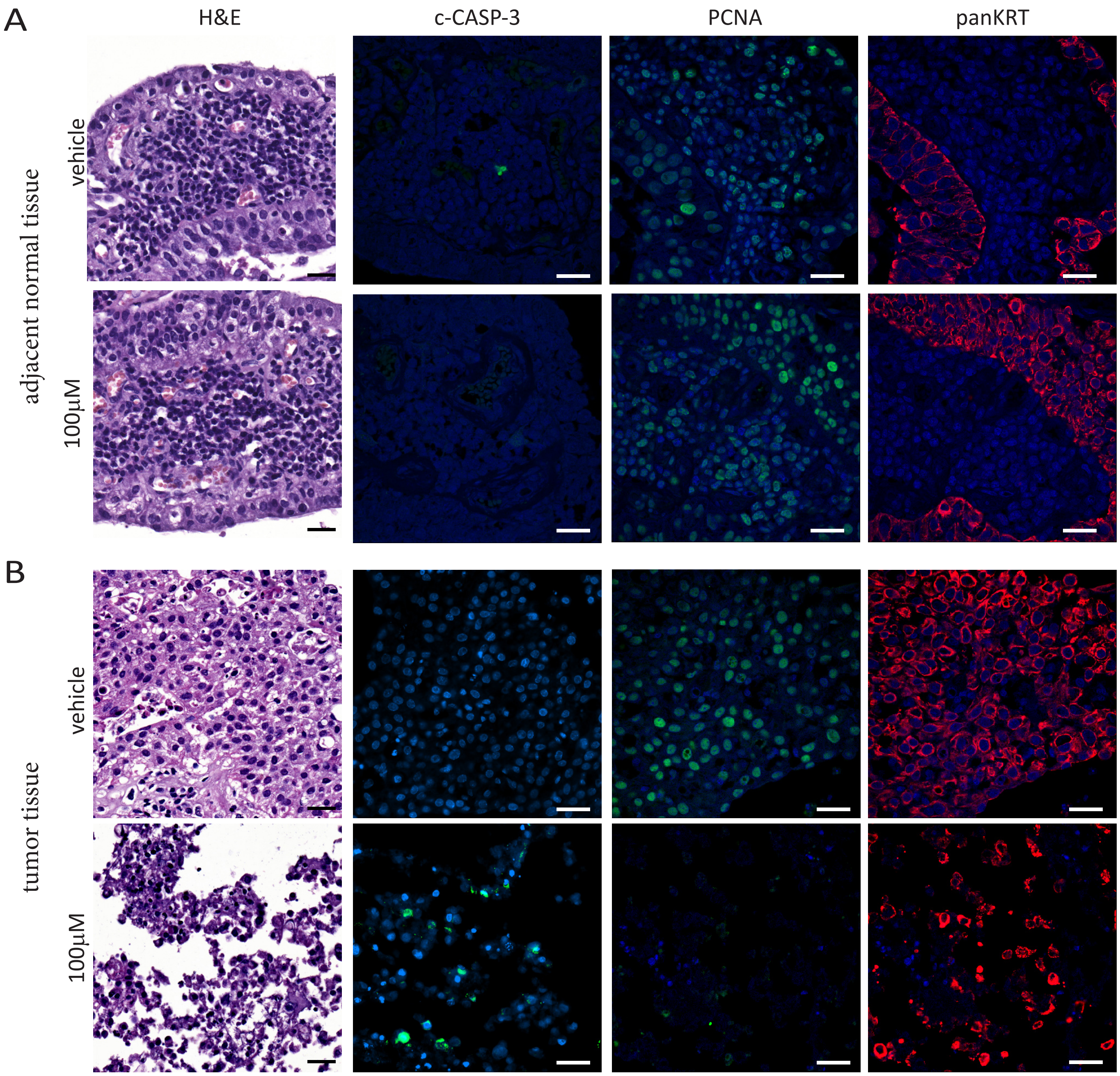

Figure S6 Evaluation of the effect of penfluridol on normal human urothelium

A) Representative images of urothelium in tissue slices obtained from adjacent normal tissue from a bladder cancer patient with NMIBC T1 grade 2 (#29). B) Representative images of tissue slices from tumor tissue from this patient (#29). H&E, c-CASP-3 and PCNA: green, panKRT red and DAPI blue. Scalebar 25µm.

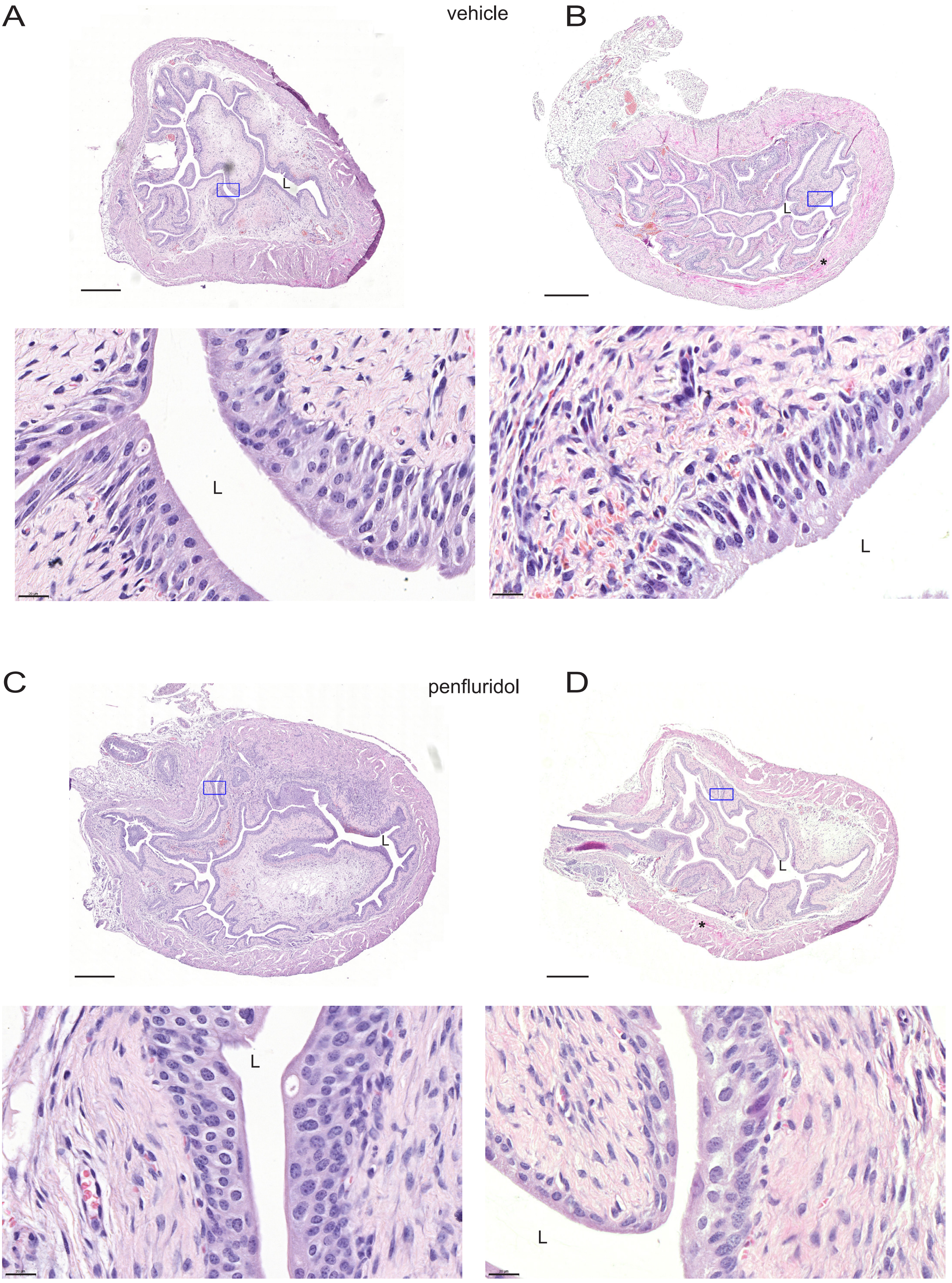

Figure S7 Evaluation of the effect of penfluridol on normal murine urothelium
A) Representative overview images of H&E stained non-tumor bearing murine bladders of vehicle-treated (A-B) and 100 µM penfluridol-treated (C-D) mice. L: lumen; * muscle layer
