# Supplementary material for: Cationic amphiphilic drugs as potential anticancer therapy for bladder cancer
Source: Mol Oncol. 2020 Oct 16;14(12):3121–34. doi: 10.1002/1878-0261.12793 (PMC7718956; doi:10.1002/1878-0261.12793)
Supplement: Supplementary file 2 — Table S1. Key resources. [file MOL2-14-3121-s002.docx]

| **RESOURCE or REAGENT** | **SOURCE** | **IDENTIFIER** | **ADDITIONAL INFORMATION** | | |
| --- | --- | --- | --- | --- | --- |
| **Experimental models: cell lines** | | | |  |  |
| CELL LINE | SOURCE | IDENTIFIER | MEDIUM | SUPPLIER | SUPPLEMENTS |
| UM-UC-3 | ATCC | ATCC Cat# CRL-1749, RRID:CVCL_1783 | Eagle's minimal essential medium (EMEM) | ATCC, 30-2003 | 10% FBS , 100 units/ml penicillin, 50 μg/ml streptomycin |
| J82 | ATCC | ATCC Cat# HTB-1, RRID:CVCL_0359 | Dulbecco’s Modified Eagle medium (DMEM) | Life technologies, Gibco, 31966-021 | 10% FBS , 100 units/ml penicillin, 50 μg/ml streptomycin |
| T24 | ATCC | ATCC Cat# HTB-4, RRID:CVC L_0554 | RPMI 1640 | Lonza, BE12-167F | 10% FBS , 100 units/ml penicillin, 50 μg/ml streptomycin, GLUTAMAX |
| TCCSUP | ATCC | ATCC Cat# HTB-5, RRID:CVCL_1738 | Eagle's minimal essential medium (EMEM) | ATCC, 30-2003 | 10% FBS , 100 units/ml penicillin, 50 μg/ml streptomycin |
| RT-112 | Cell lines service. https://clsgmbh.de | CLS Cat# 300324/p10280_RT-112, RRID:CVCL_1670 | Dulbecco’s Modified Eagle medium (DMEM) | Life technologies, Gibco, 31966-021 | 10% FBS , 100 units/ml penicillin, 50 μg/ml streptomycin |
| 5637 | ATCC | ATCC Cat# HTB-9, RRID:CVCL_0126 | RPMI 1640 | Lonza, BE12-167F | 10% FBS , 100 units/ml penicillin, 50 μg/ml streptomycin, GLUTAMAX |
| RT-4 | ATCC | ATCC Cat# HTB-2, RRID:CVCL_0036 | McCoy’s 5A | Thermo Fisher Scientific Invitrogen 36600-021 | 10% FBS , 100 units/ml penicillin, 50 μg/ml streptomycin, GLUTAMAX |
| **Experimental models: organisms/strains** | | |  |  |  |
| STRAIN | SOURCE | IDENTIFIER | GENDER | ETHICAL COMMITEE | ETHICAL PROTOCOL |
| Female balb c nude mice (CAnN.Cg-Foxn1nu/Crl) | Charles River Laboratories RRID:SCR_003792 | RRID:MGI:2160479 | FEMALE | Committee on the Ethics of Animal Experiments of Leiden University, The Netherlands | DEC_14212 |
| **Experimental models: biological samples** | | |  |  |  |
| BIOLOGICAL SAMPLES | SOURCE | IDENTIFIER |  |  |  |
| Human bladder samples transurethral resection of the bladder (TURB) | Erasmus Medical Center bladder cancer biobank | Coded samples |  |  | MEC-2014-553 |

| **RESOURCE or REAGENT** | **SOURCE** | **IDENTIFIER** | **ADDITIONAL INFORMATION** | | |
| --- | --- | --- | --- | --- | --- |
| **Antibodies** |  |  |  |  |  |
| ANTIBODY | SOURCE | IDENTIFIER | DILUTION | PC |  |
| Mouse-anti human Proliferating cell nuclear antigen (PCNA) | Sigma-Aldrich, P8825 | RRID:AB_477413 | 1:2000 | 40’ |  |
| Rabbit anti-human Pan cytokeratin (panKRT) | Life technologies, 180059 | RRID:AB_10942225 | 1:250 | 40’ |  |
| Guinea Pig anti-murine Pan cytokeratin (panKRT) | Origene, BP5069 | RRID:AB_979529 | 1:250 | 40’ |  |
| Goat anti-human Collagen I | Southerenbiotech, 1310-01 | RRID:AB_2753206 | 1:1000 | 40’ |  |
| Rabbit anti-human Cleaved caspase-3 (c-CASP-3) | Cell signaling, 9661L | RRID:AB_2341188 | 1:500 | 40’ |  |
| Mouse anti-human Keratin 18 (KRT18) | Dako, M7010 | RRID:AB_2133299 | 1:500 | 40’ |  |
| Mouse anti-human LAMP-1 | Abcam ab25630 | RRID:AB_470708 | 1:20 | N.A. |  |
| Rabbit anti-human Galectin-1 (LGALS1) | AbCaM ab25138 | , RRID:AB_2136615 | 1:2000 | N.A. |  |
| Donkey-anti Mouse Alexa Fluor 488 | Life technologies, R37114 | RRID:AB_2556542 | 1:250 | N.A. |  |
| Donkey-anti Rabbit Alexa Fluor 488 | Life technologies, A-21206 | RRID:AB_141708 | 1:250 | N.A. |  |
| Donkey-anti Mouse Alexa Fluor 555 | Life technologies, A-31570 | RRID:AB_2536180 | 1:250 | N.A. |  |
| Donkey-anti Rabbit Alexa Fluor 555 | Life technologies, A-31572 | RRID:AB_162543 | 1:250 | N.A. |  |
| Donkey-anti Goat Alexa Fluor 647 | Life technologies, A-31572 | RRID:AB_141844 | 1:250 | N.A. |  |
| Goat-anti Guinea Pig Alexa Fluor 647 | Life technologies, A-21450 | RRID:AB_2735091 | 1:250 | N.A. |  |
|  |  |  |  |  |  |

|  |  | |  |  |  |  |
| --- | --- | --- | --- | --- | --- | --- |
| **RESOURCE or REAGENT** | **SOURCE** | | **IDENTIFIER** | **ADDITIONAL INFORMATION** | | |
| **Chemicals** |  | |  |  |  |  |
| CHEMICAL | SOURCE | | IDENTIFIER |  |  |  |
| Penfluridol | Sigma-Aldrich RRID:SCR_008988 | | P3371 |  |  |  |
| Astemizole | Sigma-Aldrich RRID:SCR_008988 | | A2861 |  |  |  |
| Terfenadine | Sigma-Aldrich RRID:SCR_008988 | | T9652 |  |  |  |
| Sertinidole | Sigma-Aldrich RRID:SCR_008988 | | S8072 |  |  |  |
| Chlorprothixene | Sigma-Aldrich RRID:SCR_008988 | | C1671 |  |  |  |
| Chlorpromazine | Sigma-Aldrich RRID:SCR_008988 | | C8138 |  |  |  |
| Clemastine | Sigma-Aldrich RRID:SCR_008988 | | SML0445 |  |  |  |
| Loratadine | Sigma-Aldrich RRID:SCR_008988 | | L9664 |  |  |  |
| **Critical Commercial Assays** | | |  |  |  |  |
| ASSAY | SOURCE | | IDENTIFIER |  |  |  |
| CellTiter 96® AQueous One Solution Cell Proliferation Assay | Promega RRID:SCR_006724 | | G3581 |  |  |  |
| RealTime-Glo™ Annexin V Apoptosis and Necrosis Assay | Promega RRID:SCR_006724 | | JA1012 |  |  |  |
| **Critical equipment and software** | |  |  |  |  |  |
| EQUIPMENT/SOFTWARE | SOURCE | | IDENTIFIER |  |  |  |
| Celígo® Imaging Cytometer | Nexcelom Bioscience | | - |  |  |  |
| SpextraMax iD3 | MolecularDevices | | - |  |  |  |
| Image J version 1.48v | https://imagej.net/ | | RRID:SCR_003070 |  |  |  |
| Confocal TCS_SP8 | Leica Microsystems RRID:SCR_008960 | | - |  |  |  |
| IVIS Lumina Imaging System | Caliper LifeSciences, USA | | - |  |  |  |
| MIDI slidescanner | Pannoramic | | - |  |  |  |
| Caseviewer | 3DHistech | | RRID:SCR_017654 |  |  |  |

***Supplementary table 1 key resources***

*Key reagents and resources used in the manuscript with their respective suppliers, Research Resource Identifiers (RRID) and additional information. Cells were routinely cultured in a humidified incubator at 37°C and 5% CO2 and were regularly (once every 2 months) tested for mycoplasm by RT-PCR. Supplements were from Life Technologies, Gibco. For used antibodies, respective dilutions were added and the indicated time in the pressure cooker (PC) when antigen retrieval was performed by cooking the slides in unmasking solution (Vector Labs, H-3300); N.A. not applicable.*
