# Supplementary material for: Cationic amphiphilic drugs as potential anticancer therapy for bladder cancer
Source: Mol Oncol. 2020 Oct 16;14(12):3121–34. doi: 10.1002/1878-0261.12793 (PMC7718956; doi:10.1002/1878-0261.12793)
Supplement: Supplementary file 3 — Appendix S1. Supplementary materials and methods. [file MOL2-14-3121-s003.docx]

**Supplementary information**

**Cationic Amphiphilic Drugs**

Penfluridol, astemizole and terfenadine were obtained from Sigma-Aldrich^®^. Penfluridol (P3371) and terfenadine (T9652) were diluted in Absolute EtOH (EMSURE^®^) to a stock solution of 50 mM. Likewise, astemizole (A2861), sertinidole (S8072), chlorprothixene (C1671), chlorpotmazine (C8138), clemastine (SML0445) and loratadine (L9664) were diluted in dimethyl sulfoxide (DMSO; Sigma-Aldrich^®^) to a stock solution of 50 mM (supplementary table 2). Serial dilutions were established by diluting these stock solutions in the cell-specific medium of the various cell lines (supplementary table 1).

***In vitro* Viability assay**

Cells were seeded in a 96-wells plate at a concentration of 1500 single cells per well with 6 technical replicates. Cells were either treated after 24h, or cells were allowed to grow until confluency and subsequently treated for 2h with a dose range. After replacing the medium, cells were incubated at 37°C for an additional 24, 48 or 72h. Subsequently, 20µl of MTS reagent was added and after 2 hours, the OD was measured at 490nm using the VersaMax ELISA Microplate Reader.

Apoptotic nuclear condensation was evaluated in Hoechst-33342 stained cells using Olympus IX81 microscope with a 20 × Olympus objective, Scan^R automated acquisition software (version 2.3.0.5) and analysis with ImageJ (version 1.48v).

***In vitro* Clonogenicity *.***

100-single cells were seeded in a 6-wells plate. After 24h, cells were treated with a dose-range of penfluridol for 2h. After replacing medium, cells were incubated at 37°C. After ~14 days, cells were fixed with 4%PFA and stained for 10 minutes with 5% crystal-violet. The number of colonies and colony area were calculated with ImageJ software.

***In vitro* Lysosomal membrane permeabilization.**

60.000 cells were seeded on PET-coated chamber slides and left for 24h to adhere. Cells were treated with a dose-range of penfluridol for 2h. After 24h lysosomal membrane permeabilization was detected by staining paraformaldehyde-fixed cells with LGALS1 and LAMP-1 primary antibodies (supplementary table 1) at 4°C. DAPI staining was used to visualized the nuclei. Next, the slides were stained for 1.5h at RT with DxM 488 secondary antibody and mounted with prolong Gold antifade. Confocal images were taken with the Confocal TCS_SP8 (Leica).

***Near-patient* ex vivo *bladder cancer cultures.***

Tumor tissues from patients diagnosed with various stages of UCB were obtained during transurethral resection of the bladder upon informed consent (MEC-2014-553). Explanted tumor tissue slices (TS) were sectioned and cultured as previously described[14, 15].

Bladder cancer material was transported in EMEM medium at RT, transferred into Penicillin Streptomycin solution for 10 minutes and cut into consecutive tissue slices of approximately 500-1000 µm thick (34). The tissues slices were placed on nitrocellulose filter inserts (6 well filter inserts, pore size of 3 µm, Corning Costar) and cultured with EMEM, supplemented with 10% FCS and 1% Pen-Strep. The tissue was cultured in an oxygenated and sealed system. After 24 hours, the tissue slices were treated with either vehicle solution or penfluridol (depending on the amount of tissue either 100µM or a dose range). At 3 days post treatment, the tissue was fixed in 4% PFA for 1 hour at RT and transferred into a plastic cassette and stored in 70% ethanol at 4°C prior to paraffin embedding.

**Histology.**

*Haematoxylin and eosin staining (H&E)* was performed according to a standard protocol to assess general histology. For *immunofluorescence staining*, deparaffinization was performed via incubation in Histoclear (National Diagnostics, HS-200) and rehydrated by incubation in a series of decreasing concentrations of ethanol. Antigen retrieval was performed by cooking the slides in unmasking solution (Vector Labs, H-3300) in the pressure cooker. After blocking with 1% BSA (Sigma-Aldrich, A7906) for 30 minutes, the slides were incubated overnight with primary antibody at 4°C. Next, the slides were stained for 1.5 hours at RT with secondary antibody and mounted with prolong Gold antifade (Molecular probes, Thermofisher scientific, P36930) (supplementary table 1).

**Procedures Preclinical models**

*Orthotopic inoculation tumour cells*

1. Anaesthetize mouse with isoflurane (see below), induce miction by administering mild abdominal pressure.
2. Administer Temgesic according protocol. (see below)
3. Place the mouse ventrally on a cardboard piece
4. Tape the tail to the cardboard
5. Remove the catheter (Jelco I.V. Catheter Radiopaque 24G, ¾”, Smiths medical ref 4053) from the needle and put some instilla gel (Farco-PHARMA GmbH Koln, Germany) on the catheter.
6. Hold the urethra with a pair of tweezers and gently insert the catheter into the urethra. If you feel some resistance, pull back and try a different angle. The catheter should enter the urethra easily.
7. Insert 1 cm of the catheter and tape the catheter on top of the tail.
8. Rinse the bladder with 1 ml PBS (Check if the liquid has been equilibrated to room or body temperature)
9. Fill a syringe with HCl (0.1M)and place the needle on it. Inject approx. 100 ul into the bladder
10. Stir, using a back and forth motion of the syringe for 15” (1 stroke/sec) and remove the HCl.
11. Inject 100 ul KOH (0.1M) to neutralize the HCl.
12. Stir, using a back and forth motion of the syringe for 15” (1 stroke/sec) and remove the KOH
13. Inject cell suspension (5*10^6 in 35 ul PBS
14. Close the urethra by binding it off using a suture (Prolene Ethicon 6-0 13mm 3/8c). Let someone remove the catheter while binding off.
15. Remove tape
16. Keep the mouse anaesthetized with his lower body lifted slightly up for 2 hours
17. Cut suture open before ending the anaesthesia.

*Administration of Analgesia (Temgesic)*

1. Prepare temgesic solution (dosage: 0.1 mg/kg buprenorphine s.c. every 8 hours as needed)
2. Check if the liquid has been equilibrated to room or body temperature.
3. Fill the syringe with the substance that needs to be injected. Make sure there are no air bubbles in the syringe and that the needle is filled with fluid.
4. Take the animal from out of the cage.
5. Hold the scruff of the animal between thumb and forefinger and restrain the tail.
6. Disinfect the injection place with a cotton swab wetted with ethanol 70%.
7. Carefully insert the needle, between thumb and forefinger and into the center of the triangular shaped section of the scruff. Keep the needle and syringe parallel to the mouse’s head and back.
8. Carefully and slowly insert the substance by pushing down the plunger. You can feel the bulging of the substance between your fingers.
9. Carefully remove the needle from the scruff of the animal.
10. Check the injection site for liquid flowing back or blood loss. Do not return the mouse to its cage unless the injection site is clean and dry.
11. Place the animal back into the cage. Assure yourself of the proper/ normal movement of the animal
12. Check your animal for signs of pain or discomfort at least 6-8 hours after initial dose to determine if there is the need for additional analgesia

*Intravesical Instillation*

1. Anaesthesize mouse with isoflurane (see below)
2. Administer Temgesic according to protocol. (see below)
3. Place the mouse ventrally on a cardboard piece
4. Remove the catheter (Jelco I.V. Catheter Radiopaque 24G, ¾”, Smiths medical ref 4053) from the needle and put some instilla gel (Farco-PHARMA GmbH Koln, Germany) on the catheter.
5. Prior to the rinsing, the bladder will be emptied by mild abdominal massage, to induce a better contact between compound and urothelial cells.
6. Hold the urethra with a pair of tweezers and gently insert the catheter into the urethra. If you feel some resistance, pull back and try a different angle. The catheter should enter the urethra easily.
7. Insert 1 cm of the catheter and tape the catheter on top of the tail.
8. Rinse the bladder with 1 ml PBS (Check if the liquid has been equilibrated to room or body temperature)
9. Tie a suture around the urethra, but do not pull tight yet.
10. Insert the needle and inject 50 ul of the compound.
11. Tighten the suture, while a second person, first removes the needle. Directly followed by the catheter.
12. During 1 hour, the mice are regularly turned to contact the whole bladder surface with the compound.
13. After 1 hours the suture will be removed and the bladder will be emptied by mild abdominal compression.
14. Mice will be daily checked for weight, behavioural changes and blood in urine.

*Anaesthesia*

Isoflurane: induction phase: 2 – 4 %, maintenance phase: 0.25 – 2%

Airflow: induction phase: 0.8 L/min, maintenance phase: 0.4 L/min

When operation takes longer than 15min. the airflow (from DL meter) needs to be enriched with O2 till 30-40%.

*Bioluminescence measurements*

Outgrowth of spread of the tumour cells was monitored (number of measurements depending on the experiment) by whole body bioluminescent imaging (BLI) using an intensified charge-coupled device (CCD) video camera in a light-tight specimen box of the in vivo Imaging System (IVIS Lumina).

Animals were anesthetized with isoflurane and were given D-luciferin (15mg/ml in D-PBS; 25μl/10 g body weight) by intraperitoneal injection. Mice were placed into the light-tight camera box with continuous exposure to isoflurane. Imaging time ranged from 10 s to 1 min (generally 30s), depending on the tumor model, 5 min after D-luciferin injection. Three mice were imaged each time from a dorsal and a ventral view. The photons emitted from the bioluminescent tumours or cells were detected by the IVIS camera system, integrated, digitized and displayed. When saturated pictures are taken, first the F-stop is adjusted (diaphragm). If the image is still saturated, the image time can be adjusted.

*Intraperitoneal injections (luciferin)*

1. Check if the liquid has been equilibrated to room or body temperature.
2. Fill the syringe with the substance that needs to be injected. Make sure that there are no air bubbles in the syringe and that the needle is filled with fluid.
3. Take the animal from out the cage
4. Restrain the animal. Hold the animal with the belly facing up, tilting the animal in a head down position.
5. Disinfect the injection place with a gauze wetted with ethanol 70%.
6. Insert the needle, with the bevel facing up, into the animal’s lower right abdominal quadrant, avoiding the abdominal midline so that you will not inject into the urinary bladder
7. Carefully retract the plunger to check if you haven’t hit a blood vessel
8. With no blood in the syringe gently inject the substance.
9. Carefully remove the needle from the animal
10. Check the injection site for liquid flowing back or blood loss.
11. Place the animal back into the cage. Check for proper/ normal movement of the animal.

*Cervical dislocation*

1. Anaesthetize the mouse with isoflurane.
2. Place the thumb and first finger of the other hand against the back of the neck at the base of the skull
3. To produce the dislocation, quickly push forward and downward with the hand or object restraining the head, while pulling backward with the hand holding the tail base
4. The effectiveness of dislocation can be verified by feeling for a separation of cervical tissues. When the spinal cord is severed, a 2-4 mm space will be palpable between the occipital condyles and the first cervical vertebra. Occasionally, however, the dislocation occurs between thoracic vertebrae. Check closely to confirm respiratory arrest and when possible, verify by palpation, that there is no heart beat

*Quantification of signals*

Quantification of signals was performed by the Living Image® (Xenogen) software. Values were expressed as RLUs in photons/second. Numbers of metastases per animal were counted by eye from a dorsal and ventral view.
